# A complete compendium of crystal structures for the human SEPT3 subgroup reveals functional plasticity at a specific septin interface

**DOI:** 10.1107/S2052252520002973

**Published:** 2020-03-28

**Authors:** Danielle Karoline Silva do Vale Castro, Sabrina Matos de Oliveira da Silva, Humberto D’Muniz Pereira, Joci Neuby Alves Macedo, Diego Antonio Leonardo, Napoleão Fonseca Valadares, Patricia Suemy Kumagai, José Brandão-Neto, Ana Paula Ulian Araújo, Richard Charles Garratt

**Affiliations:** a Instituto de Física de São Carlos, Universidade de São Paulo, Avenida Joao Dagnone 1100, São Carlos-SP 13563-723, Brazil; b Instituto de Química de São Carlos, Universidade de São Paulo, Avenida Trabalhador São-carlense 400, São Carlos-SP 13566-590, Brazil; c Federal Institute of Education, Science and Technology of Rondonia, Rodovia BR-174, Km 3, Vilhena-RO 76980-000, Brazil; d Departamento de Biologia Celular, Universidade de Brasília, Instituto de Ciências Biológicas, Brasília-DF 70910900, Brazil; e Diamond Light Source, Diamond House, Harwell Science and Innovation Campus, Didcot OX11 0DE, United Kingdom

**Keywords:** septins, GTP binding/hydrolysis, filaments, protein structure, X-ray crystallography

## Abstract

Human septins 3, 9 and 12 are the only members of a specific subgroup of septins that display several unusual features, including the absence of a C-terminal coiled coil. This particular subgroup (the SEPT3 septins) are present in rod-like octameric protofilaments but are lacking in similar hexameric assemblies, which only contain representatives of the three remaining subgroups. Both hexamers and octamers can self-assemble into mixed filaments by end-to-end association, implying that the SEPT3 septins may facilitate polymerization but not necessarily function. These filaments frequently associate into higher order complexes which associate with biological membranes, triggering a wide range of cellular events. In the present work, a complete compendium of crystal structures for the GTP-binding domains of all of the SEPT3 subgroup members when bound to either GDP or to a GTP analogue is provided. The structures reveal a unique degree of plasticity at one of the filamentous interfaces (dubbed NC). Specifically, structures of the GDP and GTPγS complexes of SEPT9 reveal a squeezing mechanism at the NC interface which would expel a polybasic region from its binding site and render it free to interact with negatively charged membranes. On the other hand, a polyacidic region associated with helix α5′, the orientation of which is particular to this subgroup, provides a safe haven for the polybasic region when retracted within the interface. Together, these results suggest a mechanism which couples GTP binding and hydrolysis to membrane association and implies a unique role for the SEPT3 subgroup in this process. These observations can be accounted for by constellations of specific amino-acid residues that are found only in this subgroup and by the absence of the C-terminal coiled coil. Such conclusions can only be reached owing to the completeness of the structural studies presented here.

## Abstract 2

The relationship between GTP binding and hydrolysis on the one hand and septin filament assembly and function on the other has yet to be fully elucidated. The crystal structures of SEPT3 subgroup members bound to either GDP or to a GTP analogue shed light on this question by indicating how the squeezing of one of the inter-subunit interfaces may lead to the exposure of a polybasic region important for membrane association.

## 1. Introduction   

Septins are GTP-binding proteins that are involved in important cellular processes such as cytokinesis, membrane trafficking and microtubule dynamics. They also play more passive roles as scaffolds for the recruitment of cytoskeletal components, as diffusion barriers in membranes and even in the imprisonment of microorganisms. Over recent years, several excellent reviews have appeared emphasizing different aspects of septin biochemistry and cell biology (Akhmetova *et al.*, 2018 ▸; Barral & Kinoshita, 2008 ▸; Field & Kellogg, 1999 ▸; Fung *et al.*, 2014 ▸; Kinoshita, 2006 ▸; Mostowy & Cossart, 2012 ▸; Neubauer & Zieger, 2017 ▸; Spiliotis & Nelson, 2006 ▸; Weirich *et al.*, 2008 ▸). Many of these biological functions are dependent on the intrinsic ability of septins to spontaneously polymerize into filaments, which subsequently assemble into higher order structures such as rings and networks that are capable of membrane association. Our current knowledge of the 3D structure of septins and the way in which they associate into these filaments has recently been documented (Valadares *et al.*, 2017 ▸).

Most septins are characterized by three distinct structural domains: a variable N-terminal domain including a polybasic region that is capable of interacting with specific membrane components (Casamayor & Snyder, 2003 ▸; Zhang *et al.*, 1999 ▸), a central GTP-binding domain (G domain) including the so-called septin unique element (SUE; Versele & Thorner, 2005 ▸), and a C-terminal domain that normally includes heptad repeats characteristic of coiled coils (Pan *et al.*, 2007 ▸; Versele *et al.*, 2004 ▸). The latter have been shown to mediate interactions between septin monomers and are presumed to be essential for correct filament assembly (Marques *et al.*, 2012 ▸; Meseroll *et al.*, 2013 ▸; Sala *et al.*, 2016 ▸).

In humans, 13 different septins have been described and subdivided into four distinct subgroups based on sequence similarity: group I (SEPT3, SEPT9 and SEPT12), group II (SEPT6, SEPT8, SEPT10, SEPT11 and SEPT14), group III (SEPT1, SEPT2, SEPT4 and SEPT5) and group IV (SEPT7) (Cao *et al.*, 2007 ▸; Kinoshita, 2003 ▸; Martínez *et al.*, 2004 ▸; Pan *et al.*, 2007 ▸). In an alternative nomenclature (which we will adopt here) the groups are referred to by the name of a representative member: SEPT3, SEPT6, SEPT2 and SEPT7, respectively. Filaments are built by the polymerization of core complexes, or protofilaments, which may be either hexameric or octameric in nature. Octamers include representatives of each of the four groups (two copies each), whilst hexamers are similar but lack a SEPT3 subgroup septin. It has recently been shown that the order of septins within the core complexes is most likely to be SEPT2–SEPT6–SEPT7–SEPT7–SEPT6–SEPT2 for hexamers and SEPT2–SEPT6–SEPT7–SEPT9–SEPT9–SEPT7–SEPT6–SEPT2 for octamers (Mendonca *et al.*, 2019 ▸; Soroor *et al.*, 2019 ▸). These can then associate end to end to form mixed apolar filaments (Soroor *et al.*, 2019 ▸) that present two alternating interfaces known as G and NC (Sirajuddin *et al.*, 2007 ▸; Valadares *et al.*, 2017 ▸).

Each group of septins appears to present a series of unique features built into their amino-acid sequences which are presumably important for correct filament assembly. This spontaneous process is far from fully understood and is complicated by the fact that the G domains of individual septins, when crystallized, tend to form filaments in the crystal which employ the same G and NC interfaces as observed in a heterofilament, raising the intriguing question of the origin of interface selectivity (Valadares *et al.*, 2017 ▸). The SEPT3 subgroup members are particularly interesting. They possess G domains which share approximately 67% sequence identity, but are unusual in that they lack the C-terminal coiled coil. Although apparently facultative for core complex formation (since they do not participate in hexamers), they are nevertheless presumably indispensable for at least some aspects of filament functionality, albeit potentially in a nonstoichiometric ratio (Soroor *et al.*, 2019 ▸). Furthermore, SEPT3 septins show an interesting tissue distribution, in which SEPT9 is ubiquitous and presents a wide range of splice variants (Connolly *et al.*, 2014 ▸), whereas SEPT3 and SEPT12 are largely neurone- and testis-specific, respectively (Hall *et al.*, 2005 ▸). SEPT12 mutations affecting GTP binding and hydrolysis as well as site-specific phosphorylation events are related to male infertility (Kuo *et al.*, 2015 ▸; Shen *et al.*, 2017 ▸), whereas SEPT3 polymorphism is associated with susceptibility to Alzheimer’s disease (Takehashi *et al.*, 2004 ▸). Mutation in the N-terminal region of SEPT9 is related to hereditary neuralgic amyotrophy (Montagna *et al.*, 2015 ▸), and in-frame fusion with the MLL gene is associated with acute myelomonocytic leukaemia (Osaka *et al.*, 1999 ▸).

Since it became apparent that filaments are formed by septins belonging to different subgroups, each of which typically includes more than one member, many *in vivo* and *in vitro* studies have been carried out to identify filaments containing different combinations (Field *et al.*, 1996 ▸; Fujishima *et al.*, 2007 ▸; Hsu *et al.*, 1998 ▸; Kim *et al.*, 2011 ▸; Kinoshita *et al.*, 2002 ▸; Kuo *et al.*, 2015 ▸; Lukoyanova *et al.*, 2008 ▸; Martínez *et al.*, 2004 ▸, 2006 ▸; Nagata *et al.*, 2004 ▸; Sellin *et al.*, 2011 ▸; Xie *et al.*, 2007 ▸). Kinoshita identified that starting from the heterofilament composed of SEPT2, SEPT6 and SEPT7, it should be possible to replace both SEPT2 and SEPT6 by other septins belonging to their respective subgroups without compromising the formation of the heterofilament (Kinoshita, 2003 ▸). SEPT7, on the other hand, is unique and is expected to be essential for all viable combinations. Kinoshita’s hypothesis implies that septins from the same group should have similar structural characteristics in order to maintain the specific interactions that are made between monomers at the G and NC interfaces and thereby retain filament stability.

Currently, there is experimental evidence for the existence of three specific octameric filaments involving SEPT3 sub­group members. In the cases reported to date, SEPT9 and SEPT12 are believed to interact directly with SEPT7 through an NC interface (Kim *et al.*, 2011 ▸; Kuo *et al.*, 2015 ▸; Sellin *et al.*, 2011 ▸). In the most recent model for the protofilament (Mendonca *et al.*, 2019 ▸; Soroor *et al.*, 2019 ▸) this places a homodimer of such septins at the centre of the octameric particle, suggesting an important role in the maintenance of its palindromic structure (Mendonca *et al.*, 2019 ▸; Soroor *et al.*, 2019 ▸). The G domain of SEPT3 is the only structure of its type described to date (at medium resolution; Macedo *et al.*, 2013 ▸) and it presents several notable structural differences when compared with members of the remaining three subgroups. At least two of these are suggestive of functional significance. Firstly, helix α5′ (and its associated polyacidic region; Valadares *et al.*, 2017 ▸) lies in a different orientation to that observed in other septins, roughly parallel to the filament axis. Secondly, it is the only structure that presents a ‘closed’ NC interface, in which two monomers are squeezed together, leading to a significant rearrangement of the inter-subunit interactions involved. Nevertheless, it is currently unknown whether these structural features are interrelated and/or whether they are common to all members of the SEPT3 subgroup.

Here, we describe the crystal structures of all three septins of the SEPT3 subgroup (SEPT3, SEPT9 and SEPT12) complexed with either a GTP analogue (GTPγS or GMPPNP) or with GDP itself. Interestingly, the structure of the SEPT9 complex with GTPγS was obtained by soaking GDP-bound crystals with an excess of the new ligand. This is the first time that such an approach has been applied to septins. Results from biophysical studies, including oligomerization-state determination and GTP-hydrolysis activity, indicate that SEPT9 and SEPT12 present a broadly similar behaviour to that observed for SEPT3, as expected. Detailed comparative analysis of the crystal structures of these septins allows us to describe the similarities and differences between them and also reveals that the NC interface between two such septins is remarkably flexible, showing at least three significantly different packing arrangements. On the other hand, their conserved structural features shed light on Kinoshita’s proposal for substitutability between septins from the same subgroup within heterocomplexes.

## 2. Methods   

### 2.1. Expression and purification   

#### 2.1.1. Expression and purification of SEPT3   

Two different versions of recombinant SEPT3G were produced. Residues 43–330, which include the polybasic helix α_0_ (named SEPT3α0G), and residues 59–330, which lack it (SEPT3G), were obtained using the same method for expression and purification as described previously (Macedo *et al.*, 2013 ▸).

#### 2.1.2. Cloning, expression and purification of SEPT9GC and SEPT12G   

To construct the expression vectors for SEPT9GC (residues 279–568) and SEPT12G (residues 47–320), both DNA coding regions were independently cloned into the pET-28a(+) vector in frame with the His-tag coding region. *Escherichia coli* Rosetta (DE3) cells were used as the host strain for protein expression, specific details of which are given in Table 1 ▸. Briefly, cells harbouring the expression plasmids were grown whilst shaking at 37°C in LB medium supplemented with kanamycin (50 µg ml^−1^) and chloram­phenicol (34 µg ml^−1^). When the absorbance at 600 nm reached 0.6–0.8, the culture was cooled and protein expression was induced by the addition of isopropyl β-d-1-thiogalactopyranoside (IPTG) to a final concentration of 0.2 m*M*. After 16 h, the cells were centrifuged and suspended in lysis buffer according to the details given in Table 1 ▸. The cells were lysed by sonication and the crude extract was then centrifuged at 18 000*g* for 30 min at 4°C. The supernatant containing the recombinant protein was loaded onto a 2 ml metal-affinity column (see Table 1 ▸ for details) pre-equilibrated with lysis buffer. After the unbound proteins had been eliminated by exhaustive washing, the recombinant proteins were eluted using lysis buffer supplemented with 150 m*M* imidazole. The eluted proteins were loaded onto a Superdex 200 10/300 GL column pre-equilibrated with SEC buffer and driven by an ÄKTA purifier system. Elution was carried out in the same buffer at 4°C and the eluted fractions were analysed by 15% SDS–PAGE. For GTPase activity assays, we used the activity buffer for affinity chromatography and SEC was not employed.

### 2.2. GTP hydrolysis by recombinant SEPT9GC and SEPT12G   

The presence of nucleotide bound to the purified proteins and their ability to hydrolyse GTP were determined by the release of the nucleotide from the protein by chemical de­naturation with perchloric acid, following the method developed by Seckler *et al.* (1990 ▸). To remove precipitated proteins, the samples were initially centrifuged at 16 000*g* for 15 min at 4°C. The resulting supernatant was analysed at room temperature by anion-exchange chromatography (Protein-Pak DEAE 5 PW) on a Waters Alliance 2695 HPLC with detection at 253 nm. The column was equilibrated in 25 m*M* Tris–HCl pH 7.8, and 200 µl of each sample was eluted using a linear NaCl gradient (0.1–0.45 *M* over the course of 10 min). The retention times of each guanine nucleotide (GTP and GDP) were determined using 200 µ*M* GTP for SEPT9GC and 20 µ*M* GTP for SEPT12G in the sample buffer. For GTP-hydrolysis analysis, 20 µ*M* SEPT9GC (20 ml) and 15 µ*M* (10 ml) SEPT12G were incubated at 20°C with 60 and 45 µ*M* GTP for 5 and 2 h, respectively. Aliquots were collected and immediately frozen in liquid nitrogen. Subsequently, the samples were denatured with perchloric acid and analysed following the same protocol as described above. Similar experiments for SEPT3 have been reported previously (Macedo *et al.*, 2013 ▸).

### 2.3. Crystallization, data collection, structure determination and refinement   

Crystals of SEPT3α0G and SEPT3G were obtained by the hanging-drop vapour-diffusion method. For the crystallization of SEPT3α0G, 1 µl drops of sample (2.7 mg ml^−1^ protein in the presence of 1 m*M* GDP) were mixed with 1 µl reservoir solution (100 m*M* magnesium formate, 15% PEG 3350) at 4°C. For SEPT3G, 1 µl drops of sample (2.7 mg ml^−1^ protein in the presence of 1 m*M* GMPPNP, a nonhydrolysable analogue of GTP) were mixed with 1.5 µl reservoir solution (50 m*M* magnesium acetate, 10 m*M* sodium acetate, 15% PEG 8000) at 18°C. After 20 h, the crystals were quickly transferred to a cryoprotective solution (mother liquor plus 20% PEG 200) and then flash-cooled in liquid nitrogen. X-ray diffraction data were collected on beamline I02 of Diamond Light Source (DLS), UK, at a wavelength of 0.9795 Å using a PILATUS 6M detector. The data were processed to 1.83 and 1.86 Å resolution for SEPT3α0G and SEPT3G, respectively, using *xia*2 (Winter, 2010 ▸) and *AIMLESS* (Evans & Murshudov, 2013 ▸).

Purified SEPT9GC from the size-exclusion chromatography step was concentrated to 2.4 mg ml^−1^ and crystallized by the hanging-drop vapour-diffusion method. Drops composed of 1 µl sample (2.4 mg ml^−1^ in the presence of 1.5 m*M* GDP) were mixed with 1 µl crystallization solution (24% PEG 1500, 20% glycerol) at 18°C. After 17 h, the crystals were flash-cooled in liquid nitrogen prior to data collection. The structure of SEPT9GC in complex with GTPγS was obtained by soaking using an approach described here for the first time for septins. To allow nucleotide exchange, crystals of SEPT9GC initially bound to GDP were incubated overnight at 18°C in 6 µl drops of the crystallization solution to which 7 m*M* GTPγS had been added. Soaking was performed using a sitting-drop Intelli-Plate 24-4 (Art Robbins). Subsequently, the crystals were flash-cooled in liquid nitrogen and stored appropriately for data collection.

X-ray diffraction data for both complexes of SEPT9GC were collected on the PROXIMA 1 beamline at the SOLEIL Synchrotron, Saint Aubin, France at a wavelength of 0.9801 Å using an ADSC Quantum 315r detector. The data were indexed, integrated and scaled using the *XDS* package (Kabsch, 2010 ▸), yielding resolutions of 2.1 and 2.8 Å for the GDP and GTPγS complexes, respectively. Both structures were solved by molecular replacement with *Phaser* (McCoy *et al.*, 2007 ▸). In the case of the SEPT9GC–GDP complex, the previously determined crystal structure of SEPT3α0G bound to GDP (PDB entry 4z51, as described here) was employed as the search model. This structure (PDB entry 5cyo), once refined, was subsequently used as the search model for the determination of the GTPγS-bound complex.

The crystallization assays for SEPT12G were performed using the sitting-drop vapour-diffusion method. Protein samples (2 and 4 mg ml^−1^) in SEC buffer (Table 1 ▸) were incubated with 1 m*M* GDP or GMPPNP in the presence of 5 m*M* MgCl_2_. 200 nl drops of this protein solution were used for screening with 96 different conditions at 18°C using the Morpheus crystallization kit (Molecular Dimensions). After 24 h, crystals were obtained in conditions A12 [12.5%(*w*/*v*) PEG 1000, 12.5%(*w*/*v*) PEG 3350, 12.5%(*v*/*v*) MPD; 0.3 *M* magnesium chloride, 0.3 *M* calcium chloride; 0.1 *M* bicine/Trizma base pH 8.5], G2 [10%(*w*/*v*) PEG 8000, 20%(*v*/*v*) ethylene glycol; 0.2 *M* sodium formate, 0.2 *M* ammonium acetate, 0.2 *M* trisodium citrate, 0.2 *M* sodium potassium l-tartrate, 0.2 *M* sodium oxamate; 0.1 *M* MES/imidazole pH 6.5] and D10 [10%(*w*/*v*) PEG 8000, 20%(*v*/*v*) ethylene glycol; 0.2 *M* 1,6-hexanediol, 0.2 *M* 1-butanol, 0.2 *M* (*RS*)-1,2-pro­panediol, 0.2 *M* 2-propanol, 0.2 *M* 1,4-butanediol, 0.2 *M* 1,3-propanediol; 0.1 *M* bicine/Trizma base pH 8.5] and these were flash-cooled in liquid nitrogen. Three X-ray diffraction data sets were collected on beamline I24 at Diamond Light Source. Two corresponded to SEPT12G complexed to GMPPNP (at resolutions of 1.86 and 2.12 Å) and one corresponded to the GDP complex (at 2.19 Å). The data were auto-processed using the *xia*2 pipeline. All structures were solved by molecular replacement using *Phaser* with the SEPT3α0G–GDP structure (PDB entry 4z51) as the search model.

All of the structures were refined using *Phenix* (Liebschner *et al.*, 2019 ▸). *Coot* (Emsley *et al.*, 2010 ▸) was employed for model building into σ_A_-weighted 2*F*_o_ − *F*_c_ and *F*_o_ − *F*_c_ electron-density maps. The GDP and GTPγS molecules were automatically placed using the Find Ligand routine of *Coot*, and water molecules were identified and positioned using a combination of *Coot* and *Phenix* routines. The parameters *R*_work_ and *R*_free_ were monitored in order to evaluate the validity of the refinement protocol, and the stereochemistry of the models was assessed using *MolProbity* (Chen *et al.*, 2010 ▸). The data-collection, processing and refinement statistics are shown in Table 2 ▸.

The coordinates and structure factors have been deposited in the PDB as entries 4z51 (SEPT3G–GTPγS), 4z54 (SEPT3α0G–GDP), 5cyo (SEPT9GC–GDP), 5cyp (SEPT9GC–GTPγS), 6mq9 (SEPT12G–GMPPNP), 6mqb (SEPT12G–GMPPNP) and 6mqk (SEPT12G–GDP).

### 2.4. Synchrotron-radiation circular dichroism (SRCD)   

All septin peptides corresponding to the α_0_ polybasic region were purchased from GenScript, New Jersey, USA. SRCD measurements were performed on the AU-CD beamline at ASTRID2 at the University of Aarhus, Denmark. Spectra were collected over the range 280–170 nm in 1 nm steps, as an average of three scans, at 20°C in 10 m*M* sodium phosphate buffer pH 7.4 using a short path-length (0.0097 cm) quartz Suprasil cuvette (Hellma Analytics, optical path calibrated by interferometry). All peptides were at the same concentration of 1 mg ml^−1^. *CDtool* (Lees *et al.*, 2004 ▸) was used for all SRCD data processing, including the averaging of individual scans, baseline subtraction and zeroing in the 262–270 nm region.

## 3. Results   

### 3.1. Biochemical properties of SEPT9GC and SEPT12G   

**Figure 1:**
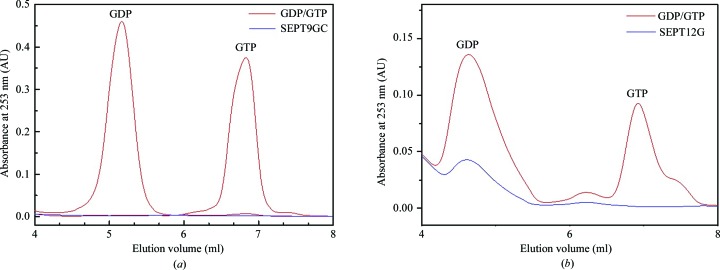
Nucleotide-content assay. (*a*) SEPT9GC, (*b*) SEPT12G. No nucleotide bound to SEPT9 was detected, indicating that SEPT9GC is purified in its apo form. However, small amounts of GDP were released from the SEPT12G sample, indicating that a fraction of the molecules were purified in the form of a GDP-bound complex. Samples of GTP and GDP [100 and 20 µ*M* of each in (*a*) and (*b*) respectively; red lines] were used as markers.

SEPT9GC and SEPT12G eluted from a Superdex 200 size-exclusion column with molecular weights of approximately 38 and 32 kDa, respectively, showing them to be monomeric in solution (data not shown). This is consistent with the results described previously for the G domain of SEPT3 (Macedo *et al.*, 2013 ▸). To evaluate whether SEPT9GC and SEPT12G purify bound to nucleotide or in the apo state, anion-exchange chromatography of the supernatant after protein denaturation was used to separate GDP from GTP. Fig. 1 ▸(*a*) shows that SEPT9GC purifies as a nucleotide-free protein, consistent with the results observed for SEPT3G (Macedo *et al.*, 2013 ▸). In contrast, SEPT12G purified with a small quantity of GDP [Fig. 1 ▸(*b*)] that was presumably acquired from the bacteria during heterologous expression.

**Figure 2:**
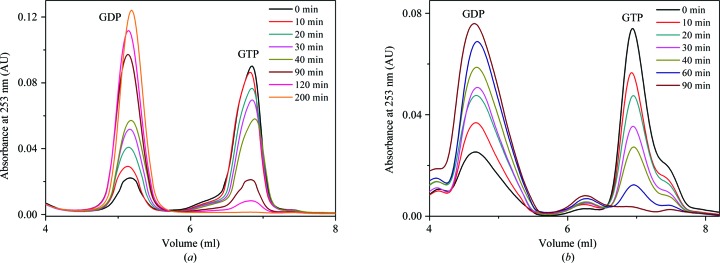
GTP hydrolysis by septins. (*a*) SEPT9GC, (*b*) SEPT12G. HPLC traces over time after incubation at 20°C with an initial GTP concentration of 60 and 45 µ*M* in (*a*) and (*b*), respectively. Both proteins show hydrolytic activity.

To verify GTP hydrolysis by SEPT9GC, a similar approach was taken in which chemical denaturation with perchloric acid was used to release nucleotide from the protein over the time course of the experiment. Apo SEPT9GC was incubated with GTP for 5 h at 20°C, and its conversion into GDP was monitored by HPLC. Under these conditions almost total conversion of GTP to GDP was observed after 200 min. Similarly, SEPT12G was able to hydrolyze all of the GTP in 90 min. These results indicate that both SEPT9GC and SEPT12G show GTPase activity, as expected (Fig. 2 ▸).

### 3.2. Septin 3 structures   

Data-collection, processing and refinement statistics are provided in Table 2 ▸ for all of the structures described.

**Figure 3:**
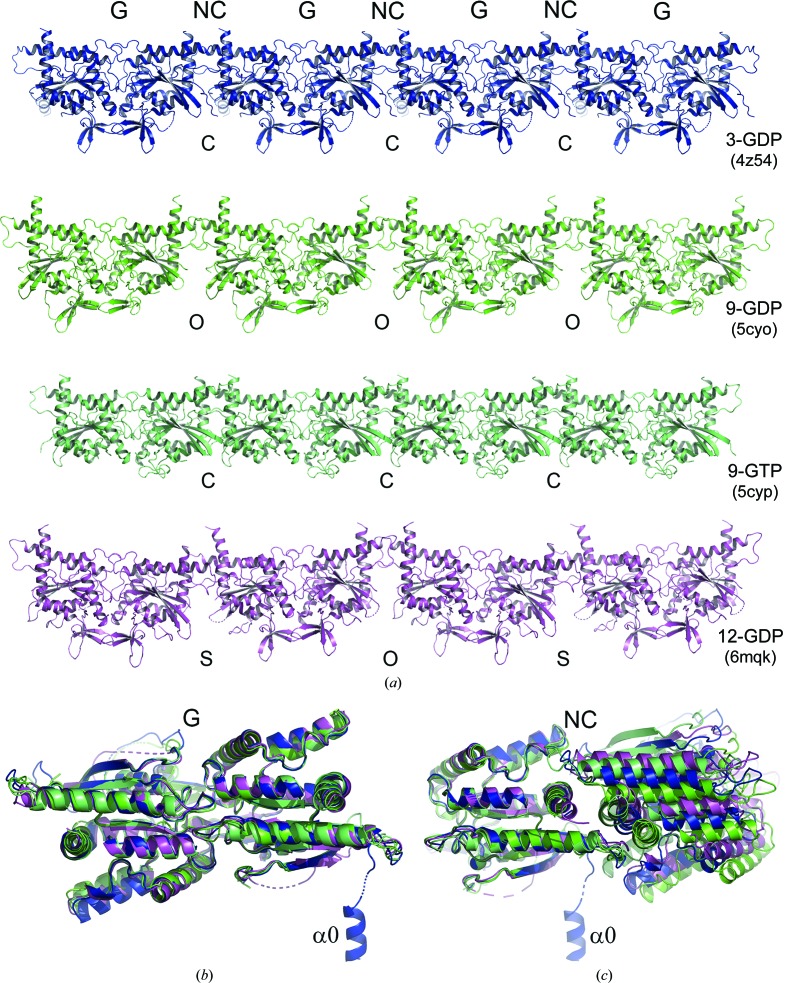
G and NC interfaces formed within the crystal structures. (*a*) Filaments observed on generating symmetry-related molecules for four representative structures. G and NC interfaces are observed in all cases, with variation occurring only at the latter. SEPT3 α0G–GDP (PDB entry 4z54) shows only closed (C) NC interfaces, SEPT9GC–GDP (PDB entry 5cyo) has only open (O) NC interfaces, SEPT9GC–GTPγS (PDB entry 5cyp) has closed interfaces which are slightly more squeezed than in SEPT3G–GDP, and SEPT12G–GDP (PDB entry 6mqk) has both open and shifted (S) NC interfaces which alternate along the filament. The filaments formed in the SEPT3G–GMPPNP complex (PDB entry 4z51) show similar interfaces to SEPT3α0G–GDP, and those formed by SEPT12G–GMPPNP (PDB entry 6mq9) are similar to those in SEPT12–GDP. (*b*) Superposition of G-interface dimers for the four structures shown in (*a*), revealing minimal structural variation. (*c*) Superposition of one subunit (left) of an NC dimer for each of the structures shown in (*a*). Considerable variation in the position of the second subunit is an indication of the different conformational states (O, C or S) of the interface. Helix α0 of the SEPT3α0G–GDP complex is specifically highlighted. The colours used for each complex will be preserved in subsequent figures.

We provide two high-resolution structures of the G domain of SEPT3 complexed with either GDP or the GTP mimetic GMPPNP (at 1.83 and 1.86 Å resolution, respectively). These structures allow a precise description of the SEPT3G fold and permit the clarification of a previously described GDP complex reported at 2.9 Å resolution (Macedo *et al.*, 2013 ▸). Despite their different space groups, in all three cases the generation of symmetry-related molecules reveals typical filaments stabilized by the characteristic G and NC interfaces. This can be seen in Fig. 3 ▸(*a*) for the GDP-bound complex reported here (PDB entry 4z54). An identical arrangement is observed in the presence of GMPPNP (PDB entry 4z51; not shown) and in the structure reported previously (PDB entry 3sop; Macedo *et al.*, 2013 ▸).

The G domain of SEPT3 has a typical septin fold (Valadares *et al.*, 2017 ▸) based on a three-layered αβα architecture, dominated by a six-stranded central β-sheet. To facilitate comprehension, Supplementary Fig. S1 shows the standard nomenclature that is used to describe the septin fold. When compared with that described previously (PDB entry 3sop), the structure reported here for the GDP complex (PDB entry 4z54) provides an accurate and unambiguous description of the Mg^2+^ ion in the active site, where it is coordinated by the side chains of Ser75 and Thr102 (from switch I), the β-phosphate of GDP and three water molecules, one of which is held by Asp125 from switch II. This gives rise to the characteristic octahedral coordination of the metal. The asymmetric unit contains two independent copies of the SEPT3α0G construct, and several regions are more complete than in the previously reported structure. These include switch I and the β-turn between strands 2 and 3 (in subunit *A*) as well as switch II and helix α5′ (in both subunits). Switch II forms the canonical antiparallel β-bridge across the intersubunit interface, as described recently by Brognara *et al.* (2019 ▸). The highly distorted three-stranded β-meander (β9, β10/β7 and β8), which contributes to the G interface and is separate from the main β-sheet, is complete in both subunits of SEPT3α0G–GDP (PDB entry 4z54).

The SEPT3α0G construct includes the polybasic region (PB1) prior to the GTP-binding domain. This region has been reported to form a short domain-swapped α-helix (α0) in both the SEPT2–SEPT6–SEPT7 heterocomplex (PDB entry 2qag) and in the SEPT2 G domain alone (PDB entry 2qa5) (Sirajuddin *et al.*, 2007 ▸). In both cases it nestles within the NC interface, where it is stabilized by its proximity to the polyacidic region which precedes helix α5′. In the SEPT3α0G–GDP structure reported here the α0 helix is only well ordered in the *B* subunit, where it occupies a very different orientation to that previously described and the PB1 region connects it to the first β-strand (β1). It splays outwards from the filament [Figs. 3 ▸(*b*) and 3 ▸(*c*)] and appears to be stabilized by fortuitous inter­actions principally with a cavity formed by the α6, α2 and α5 helices of crystallographically related filaments. Allied to the fact that the short ordered segment of the same region in the *A* subunit (Lys53–Gly59) takes a completely different course, this suggests that the α0 helix is highly flexible when not anchored within the NC interface.

Septins have a second polybasic region (PB2) following helix α2 (Omrane *et al.*, 2019 ▸). In the low-resolution structure previously reported (PDB entry 3sop) this was described as adopting a completely novel conformation differing from that observed in all other known septin structures. This is borne out here in the high-resolution structure of SEPT3α0G–GDP. As a consequence, PB2 lies close to the polyacidic region of a neighbouring subunit, with the electrostatic interactions occurring across an NC interface which will be described more fully below.

The SEPT3G–GMPPNP complex (PDB entry 4z51) has only one monomer per asymmetric unit, which superposes well with either monomer of the SEPT3α0G–GDP structure, resulting in a mean r.m.s.d. of 0.37 Å for 251 C^α^ atoms. No major differences were therefore observed between the two structures, besides the fact that the construct used for the GMPPNP complex lacks PB1 (helix α0). Switches I and II are partially and completely ordered, respectively, and the only difference in the Mg^2+^ coordination is that one of the water molecules has been replaced by an O atom from the γ-phosphate.

The presence of GMPPNP bound to SEPT3G allows a precise description of the ligand-binding site, particularly the interactions made via the γ-phosphate, which have not been described previously. Supplementary Fig. S2 shows a schematic diagram of the interactions made by both GDP and the GTP analogue. The γ-phosphate of GMPPNP forms direct hydrogen bonds to residues from the P-loop (Ser70 and Lys74), switch I (Lys101 and Thr102) and switch II (Gly128) as well as providing a ligand to the Mg^2+^ ion. Gly128–Asp131 from switch II assume two different conformations in the different subunits of the SEPT3α0G–GDP structure. We call these the *buried* and *flipped* conformations, respectively, with reference to the orientation of the side chain of Phe129. In the former the phenylalanine side chain forms part of a buried aromatic cluster and in the latter it is flipped out of the hydrophobic core owing to changes in both the main-chain and side-chain torsion angles. By comparison, the single monomer of the GMPPNP complex has the *buried* conformation. Indeed, a strong hydrogen bond formed between the γ-phosphate and the amide N atom of Gly128 (switch II) would appear to favour this arrangement, an observation that is reinforced when examining the structures of SEPT9 described below. In small GTPases this interaction forms part of the universal switch mechanism (or ‘loaded spring’) which couples γ-phosphate release on hydrolysis to conformational change (Vetter & Wittinghofer, 2001 ▸). The remaining interactions made by GMPPNP are essentially the same as those described previously for SEPT3–GDP (PDB entry 3sop) but with additional water molecules visible owing to the improved resolution. The water structure is essentially identical to that described for the GTP complex of the catalytically inactive SEPT10 from *Schistosoma mansoni* (*Sm*SEPT10; Zeraik *et al.*, 2014 ▸).

### 3.3. Septin 9 structures   

As for SEPT3, here we describe two structures of complexes of the G domain of SEPT9, in this case bound to either GDP or the GTP analogue GTPγS. These were solved at 2.0 and 2.9 Å resolution, respectively. Unusually, the SEPT9GC–GTPγS complex was obtained by soaking pre-formed crystals of SEPT9GC–GDP.

The structure of SEPT9GC–GDP (PDB entry 5cyo) has a G-interface dimer in the asymmetric unit, with the GDP making essentially the same contacts within the active site as those described above for SEPT3. The individual monomers show differences, most of which are owing to variable structural disorder. Overall, the *B* subunit is less well ordered than the *A* subunit. For example, switch I is almost complete in subunit *A* but is much less so in subunit *B*, where 11 residues could not be modelled. Similarly, the β2–β3 hairpin loop is complete in the former but is lacking four residues in the latter. Both subunits present some degree of disorder in the region of the β-meander (β9, β10/β7 and β8) and there is a conformational difference at the junction of α5 with α6 which is justified by the electron density. Phe91 (the homologue of Phe128 in SEPT3) assumes different rotamers in the two monomers, one of which is the *buried* conformation described above. The other is an alternative, also buried, conformation which affects the side chain of Cys100 and the N-terminus of helix α2. Once again the absence of the γ-phosphate of GTP and the consequent lack of a direct hydrogen bond to switch II appears to increase the conformational freedom of the region, including Phe91.

The SEPT9GC–GTPγS complex has four monomers in the asymmetric unit and there are slight variations in the degree of disorder in several regions of the different chains. These include segments which often present variation from one septin to another [the N-terminal region, the hairpin connection between β2 and β3, switch I and the distorted β-meander (β9, β10/7 and β8)]. The latter region shows some degree of order only in subunit *A* and is most incomplete in subunits *B* and *D*. The lack of readily interpretable density in this region is the cause of apparent gaps in the crystal packing (PDB entry 5cyp). It is presumably the weakness of these contacts which allows the filaments to move within the crystal lattice on substituting GDP for GTPγS (see below). As with SEPT3, when complexed to the GTP analogue Phe91 is observed to adopt only the *buried* conformation, consistent with the presence of the hydrogen bond between switch II and the γ-phosphate.

Both complexes of SEPT9GC have a fully ordered switch II region in which the β-bridge across the G interface is well defined. The Mg^2+^ ion in the GDP complex is coordinated identically to that observed for SEPT3. However, owing to the lower resolution of the GTPγS complex, water molecules have not been included at the Mg^2+^ site. This is presumably responsible for the slight shift in the metal-ion position during refinement.

In both SEPT9GC complexes the application of crystallo­graphic symmetry generates filaments stabilized by NC and G interfaces [Fig. 3 ▸(*a*)]. However, there is a significant foreshortening of the filament in the GTPγS complex owing to closure of the NC interface, the details of which will be described below. When compared with the GDP complex (which has a canonical ‘open’ NC interface as seen in other septin subgroups), two neighbouring monomers come to­gether by approximately 8 Å. By contrast, in the case of both the GDP and GMPPNP complexes of SEPT3G the NC interface is closed.

#### 3.3.1. Cell transformation for SEPT9   

**Figure 4:**
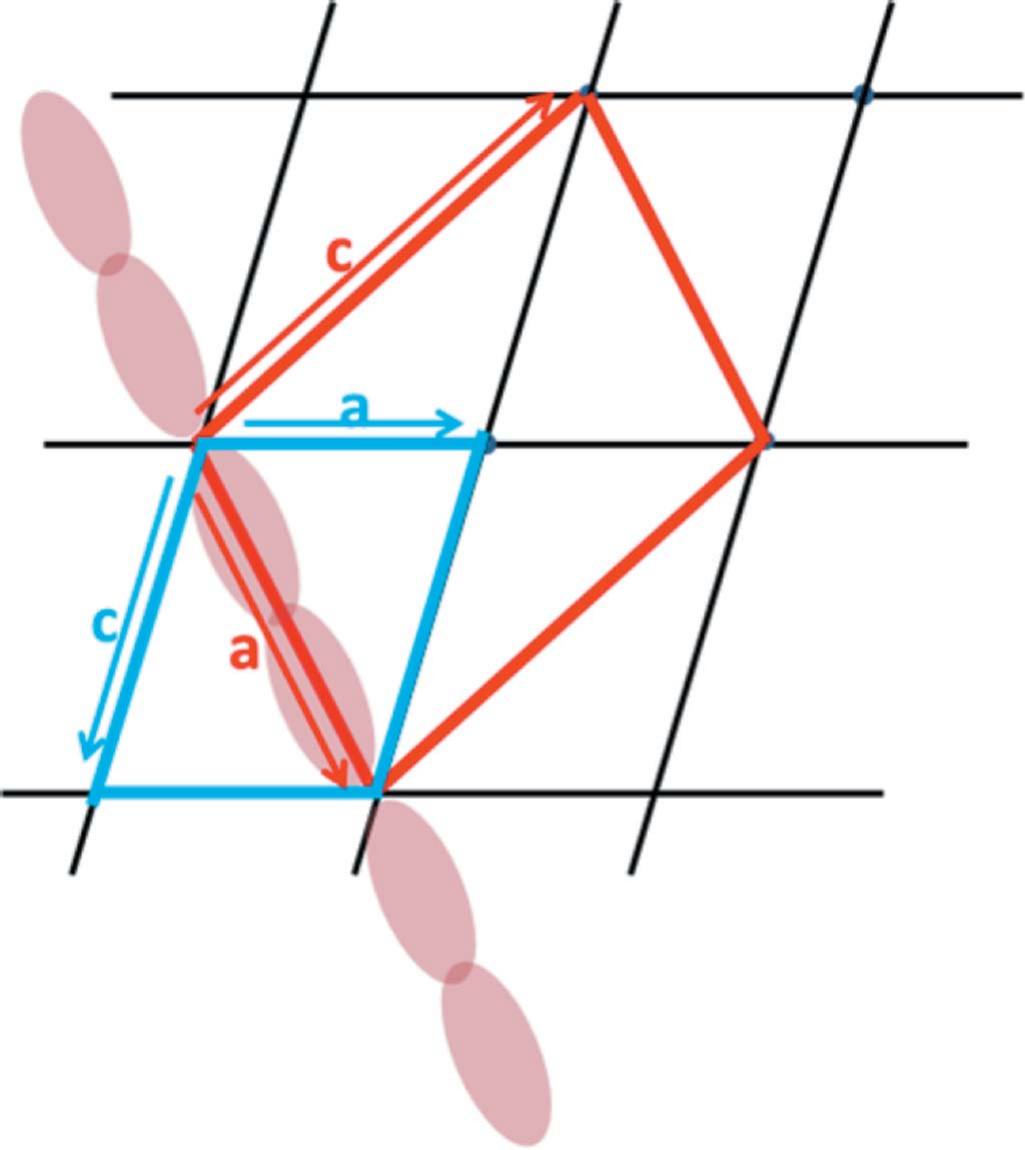
Schematic representation of the change in the unit cell in SEPT9GC complexes. GDP-bound crystals of SEPT9GC have the blue monoclinic cell with filaments running in the direction of the cell diagonal [101]. On soaking these crystals with excess GTPγS there is a unit-cell change leading to a new monoclinic cell of approximately twice the volume in which the filaments run along the *a* direction [100]. The red cell is the predicted new cell, based on the original blue cell. The *c* parameter is well predicted but *a* is foreshortened in accordance with the corresponding shrinkage of the filament which occurs on substituting GDP for GTPγS.

Given that the SEPT9GC–GTPγS complex was generated by soaking GDP-bound crystals, it is not surprising that the two crystal forms (which both belong to space group *P*2_1_) are related. In the monoclinic crystals corresponding to the GDP complex (Fig. 4 ▸) the filaments are arranged within the *ac* plane and lie parallel to the cell diagonal [101]. On the other hand, in the GTPγS complex the filaments are aligned along the *a* axis and the new monoclinic cell has approximately double the volume and twice the number of molecules per asymmetric unit. The *b* axis is common to both crystal forms but with an inverted sign. From the blue cell (*a* = 57.50, *c* = 77.44 Å, β = 105.92°) it is possible to calculate the expected cell constants for the red cell, assuming no lattice distortion. This yields 82.8 and 108.4 Å, respectively, for the new values of *a* and *c*. Whilst the *c* parameter fits well with that observed experimentally (108.22 Å), the value for *a* is overestimated by approximately 8 Å. This is consistent with the foreshortening of the filament along the *a* axis owing to the closure of the NC interface. Small rearrangements to the crystal packing also lead to a difference of approximately 7° between the predicted and observed values of β for the GTPγS-bound form.

### 3.4. Septin 12 structures   

As for the previous two cases, we report here complexes of SEPT12G bound to both GDP and to a GTP analogue. Two structures were obtained in the presence of GMPPNP (in space groups *P*1 and *C*222_1_ at 1.8 and 2.12 Å resolution, respectively) and one with GDP (in space group *P*1 at 2.19 Å resolution). Both of the *P*1 structures are isomorphous and have four monomers in the asymmetric unit, whereas the *C*222_1_ structure has only one. The individual monomers are very similar, with r.m.s.d. values ranging from 0.21 Å for different monomers from a given structure to 0.49 Å for monomers from different structures. Comparisons with the different SEPT3 and SEPT9 structures yield typical r.m.s.d. values of the order of 0.7 Å indicative of great structural conservation within the subgroup.

The guanine nucleotide-binding site of SEPT12 is well conserved when compared with the remaining subgroup members (SEPT3 and SEPT9) as well as with human septins in general. The β-phosphate of GDP is anchored by the Mg^2+^ ion together with Gly59, Leu60, Lys62, Ser63 and His170, the latter from a neighbouring subunit across the G interface. In structures that contain the GTP analogue the γ-phosphate is anchored by the Mg^2+^ ion, one water molecule and Ser58 and Lys62 from the P-loop, Thr89 from switch I and Gly115 from switch II. Arg195 replaces the lysine which is normally present, but its side chain continues to pack against the guanine base in a similar fashion. As for SEPT3 and SEPT9, all three of the SEPT12 structures possess Mg^2+^ bound in the active site, independent of the nucleotide present. However, this may not be physiologically realistic in the case of the GDP complex. In SEPT12G–GDP (PDB entry 6mqk) the metal ion is canonically coordinated by Thr89, Ser63, the β-phosphate and three water molecules (one of which is held by Asp112). The resulting binding site is effectively identical to that shown for SEPT3 in Supplementary Fig. S2.

Switch I shows some degree of disorder in almost all of the SEPT12G monomers, being complete only in the SEPT12G–GMPPNP complex in space group *C*222_1_ (PDB entry 6mqb). Switch II, on the other hand, is well ordered in all structures and forms the antiparallel β-bridge structure across the G interface as mentioned above for both SEPT3 and SEPT9. Furthermore, Phe116 (the homologue of Phe128 in SEPT3) is always observed in the *buried* conformation. Both SEPT12G–GDP and SEPT12G–GMPPNP (space group *P*1) form filaments within the crystal lattice, employing the NC and G interfaces. In contrast, SEPT12G–GMPPNP (space group *C*222_1_) does not. Rather, the monomer of the asymmetric unit forms a G-interface dimer by the application of crystallo­graphic symmetry but this does not extend into filaments via NC interfaces. For this reason, all future discussion will refer to the *P*1 structure.

The filaments of the GDP and GMPPNP complexes are effectively identical. However, they both show an unusual feature with respect to those of SEPT3 and SEPT9. Since both structures have four monomers in the asymmetric unit, there are two crystallographically independent NC and G interfaces. If these are dubbed NC_1_, NC_2_, G_1_ and G_2_ then they will alternate in the following manner along the filament: –NC_1_–G_1_–NC_2_–G_2_–NC_1_–G_1_–NC_2_–G_2_–. Whilst the two independent G interfaces are very similar, the NC interfaces are not [Fig. 3 ▸(*a*)]. One of them is in the classical ‘open’ conformation seen in the remaining septin subgroups, whilst the other is in a shifted closed conformation. In this case, rather than being related by a twofold perpendicular to the filament axis, the subunits are related by a screw axis, leading to an asymmetric arrangement in which the two subunits no longer make equivalent contacts with one another. The shifted closed interfaces are indicated with an S in Fig. 3 ▸(*a*).

### 3.5. A notable common structural feature   

**Figure 5:**
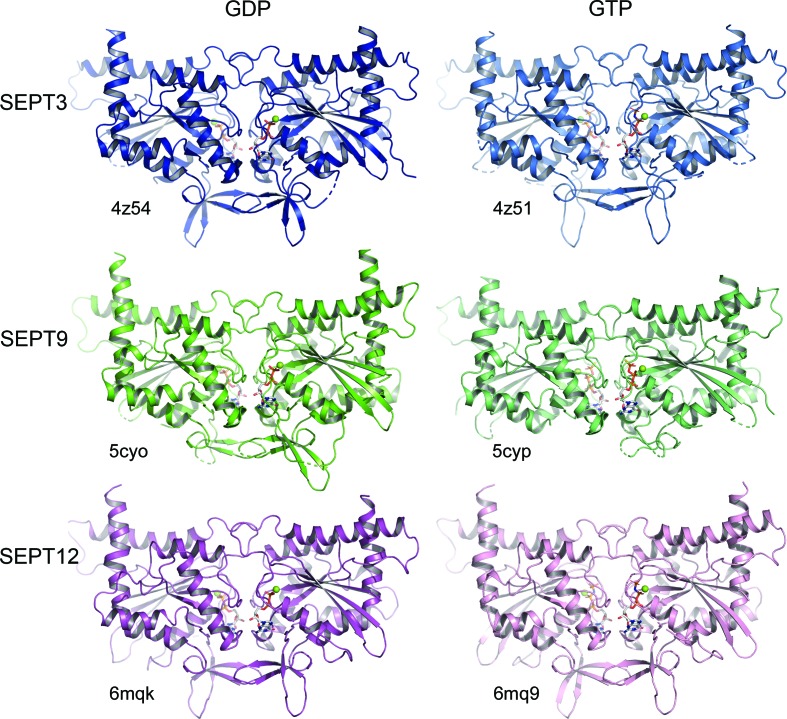
Structures of the six SEPT3-subgroup septin complexes. G-interface dimers of the GDP and GTP-analogue complexes of the G domains of SEPT3, SEPT9 and SEPT12 are shown. The colours used in this figure will be used consistently throughout this paper, in which the GDP-bound complexes are shown in a darker tone than the GTP-analogue complexes. Corresponding PDB codes are given. The lower resolution of PDB entry 5cyp means that the region of the β-meander (at the bottom of the structure) presents less well defined density which is largely uninterpretable.

**Figure 6:**
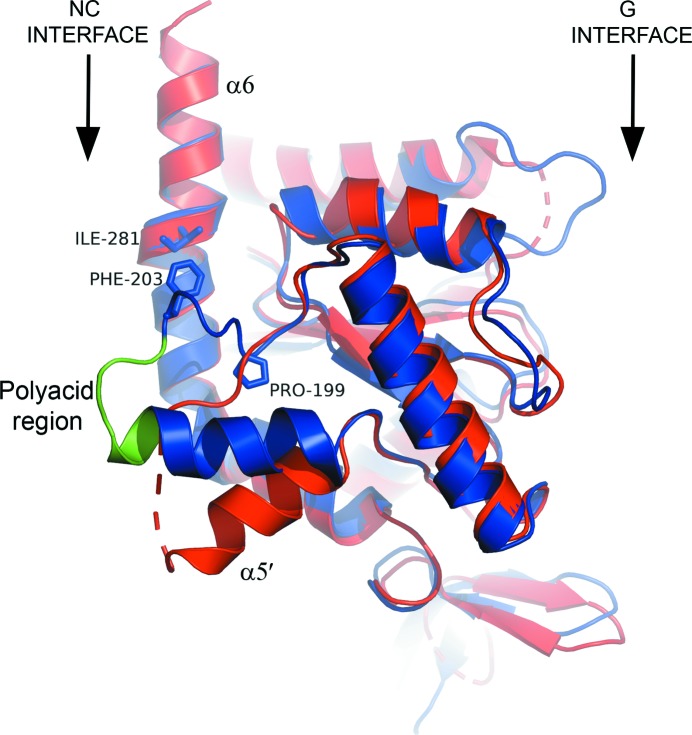
A comparison between SEPT2 and SEPT3 in the region of helix α5′. In SEPT3 (and all other subgroup members) helix α5′ is raised with respect to SEPT2 (which represents all of the remaining subgroups). This is a unique and consistent characteristic of the SEPT3 subgroup and its conformation is the result of a ‘characteristic’ proline (Pro199) which alters the course of the polypeptide chain, including the polyacidic region (green). It is maintained by the hydrophobic contact between Ile281 (from α6) and Phe203 (from the polyacidic region), both of which are also characteristic of the SEPT3 subgroup. The residue numbers used apply to SEPT3.

Fig. 5 ▸ shows the G-interface dimers for each of the six structures determined here (SEPT12G–GMPPNP in space group *C*222_1_ has been omitted in order to reduce redundancy). The similarity of the structures is immediately apparent and the highly conserved nature of the G interface is highlighted in Fig. 3 ▸(*b*), where four representative dimers have been superimposed. Nevertheless, this subgroup of septins shows some characteristic features which distinguish them from the remaining subgroups. In terms of overall fold, the most notable feature is the orientation of helix α5′, which becomes apparent on superposing SEPT3 on SEPT2 (PDB entry 2qnr; Structural Genomics Consortium, unpublished work) as a representative of the remaining three subgroups (Fig. 6 ▸). Helix α5′ lies roughly parallel to the filament axis in the SEPT3 subgroup, whereas it is noticeably inclined at approximately 45° in all others. Although this was commented on previously for SEPT3 (Macedo *et al.*, 2013 ▸), it is now apparent that it is a characteristic feature of the whole subgroup and does not vary as a function of the type of nucleotide bound. The consequence of this feature is that the polyacidic region is raised in such a way as to favour interaction with PB2 of its NC partner (see below).

### 3.6. The G and NC interfaces of the SEPT3 subgroup   

The G and NC interfaces alternate along the six filamentous structures described here (only SEPT12G–GMPPNP in space group *C*222, PDB entry 6mqb, does not form filaments). As mentioned above, all of these present the canonical G interface, of which the nucleotides bound to each subunit are an integral component. In all structures, a histidine from the β4/α3 loop (His183 in SEPT3) reaches across the G interface to interact with the β-phosphate of the neighbouring subunit. The inter-subunit salt bridge, which is a characteristic feature of septins (Glu216 and Arg280 in SEPT3) and which lies underneath the guanine base, is also present. On the other hand, in all members of the SEPT3 subgroup an otherwise conserved Glu from the P-loop has been substituted by Gln. Despite the absence of the formal negative charge, this glutamine from both subunits (Gln69 in SEPT3) participates in an interfacial cluster involving water molecules and Arg186 (SEPT3), or its homologue, in a manner similar to the glutamic acid.

The current model for the assembly of an octamer-based heterofilament (Mendonca *et al.*, 2019 ▸; Soroor *et al.*, 2019 ▸) implies that members of the SEPT3 subgroup would be expected to form a heterotypic G interface with SEPT7 and not the homotypic interface observed here. This is therefore likely to be a ‘promiscuous’ interaction as seen in many other septin crystal structures. Nevertheless, recent evidence suggests that a homotypic G interface formed by SEPT9 may be physiologically important in the control of microtubule dynamics (Nakos *et al.*, 2019 ▸).

**Figure 7:**
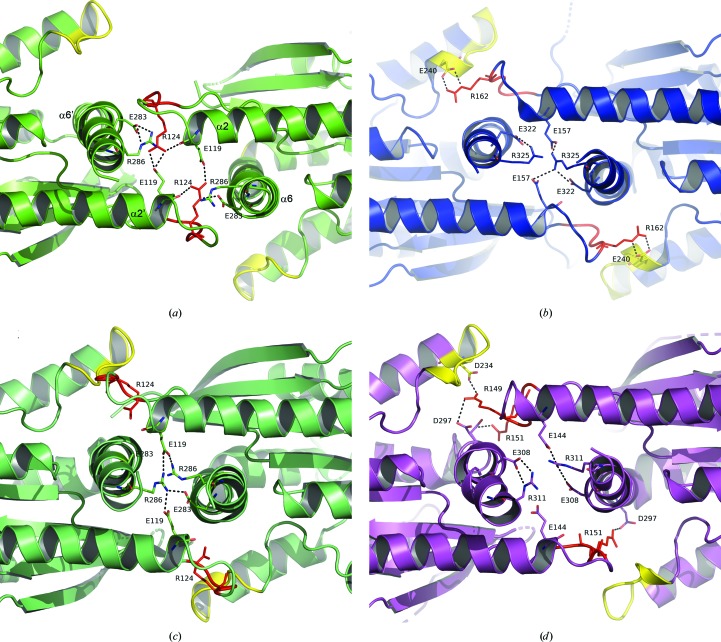
Details of the different NC interfaces. (*a*) SEPT9GC–GDP (PDB entry 5cyo) has a canonical open interface in which the PB2 region (red) does not interact with the polyacidic region (yellow) of the neighbouring subunit. The typical salt bridges involving α6 and the loop following α2 (including Arg124 from PB2) are observed (Valadares *et al.*, 2017 ▸). α2′ and α6′ refer to equivalent helices from the other subunit. (*b*) The closed interface of SEPT3α0G–GDP (PDB entry 4z54) shows how the PB2 region now interacts with the polyacidic region. In this structure several salt bridges between the two regions are present, but only that involving Arg162 (the homologue of Arg124 in SEPT9) is represented explicitly. There is significant rearrangement of the remaining salt bridges as a result of interface closure. (*c*) The closed interface of SEPT9GC–GTPyS (PDB entry 5cyp) also shows the close proximity of PB2 and the polyacidic region as a result of interface closure. In this case the subunits are slight closer together than shown in (*b*) and the poorer resolution prohibits a complete description of the interactions involved. (*d*) The shifted NC interface as observed in SEPT12G–GDP (PDB entry 6mqk). Owing to the shift of one subunit with respect to the other in a direction roughly parallel to helix α6, the interface no longer has twofold symmetry and the interactions observed on the upper side of the interface (involving PB2 and the polyacidic region) are missing on the lower side.

The NC interface in the SEPT3 subgroup presents a much more interesting and variable behaviour. By superimposing only one subunit of an NC dimer the structural variation of the interface becomes apparent [Fig. 3 ▸(*c*)]. Broadly speaking, the relative arrangement of the monomers can be classified into three types: open (considered ‘canonical’) in which the monomers are positioned further apart, closed and shifted. The open interface is observed in SEPT9GC–GDP and in one of the two types of NC interface (NC_1_) observed in both SEPT12G–GDP and SEPT12G–GMPPNP. The closed interface is present in both complexes of SEPT3 and in SEPT9GC–GTPγS, where it is even more closely packed. Finally, the shifted arrangement (which is also closed) is asymmetric and is seen only in the SEPT12 complexes, where it is present as the second type of NC interface (NC_2_). In crystal structures of other septin subgroups no such plasticity is observed; rather, all present the ‘canonical’ open interface [Fig. 7 ▸(*a*)]. Therefore, the closed conformation appears to be a unique feature of the SEPT3 subgroup alone.

The alterations which occur at the NC interface are largely owing to rigid-body movements of the individual subunits. These can be quantified by calculating the r.m.s.d. on superposing the NC dimers and comparing them with the corresponding values for the monomers. Overlaying a single monomer from the different NC interfaces observed for SEPT9GC and SEPT12G yields values of 0.57 and 0.22 Å, respectively. A similar value of 0.37 Å is observed for SEPT3G, in which the interface is always observed to be closed. Overall, therefore, there is little alteration to the structure of the monomers. However, on simultaneously superposing both monomers across the different NC interfaces we observe values of 4.66 Å for the two forms of SEPT9 (open against closed) and 4.01 Å for SEPT12 (open against shifted), indicating significantly different relative positions for the monomers in the different dimeric states. By contrast, the two forms of SEPT3 (both of which are closed) have an r.m.s.d. of only 0.48 Å, barely different from that of an individual monomer. Overall, these values are in accordance with our visual classification of the interfaces.

Examples of the detailed interactions present in each type of interface are shown in Fig. 7 ▸. In the canonical open interface as observed in the SEPT9GC–GDP complex, salt bridges involving residues from the C-terminal α6 helices and the loops following α2 (Glu119, Arg124, Glu283 and Arg286) are responsible for the stability of the interface [Fig. 7 ▸(*a*)]. These are identical to those seen in SEPT2, SEPT7 and *Sm*SEPT10 (Brognara *et al.*, 2019 ▸; Sirajuddin *et al.*, 2009 ▸; Zeraik *et al.*, 2014 ▸). In the SEPT9GC–GTPγS structure and in both SEPT3 complexes the NC interface is closed [Figs. 7 ▸(*b*) and 7 ▸(*c*)] and the electrostatic interactions, involving the same residues, are substantially rearranged. On closure, the α6 helices from both subunits come closer together whilst the α2 helices move further apart. As a consequence, the loop following α2 embraces α6 of the other subunit, bringing the PB2 region (which is most basic in the SEPT3 subgroup) into close proximity to the polyacidic region of its neighbour [Figs. 7 ▸(*b*) and 7 ▸(*c*)].

On comparing the closed conformation for SEPT9 with that observed in SEPT3, subtle differences are observed. The closure of the NC interface is of the order of 8.5 Å for SEPT9 (using the β carbons of Phe282 and His282 as markers) but about 4 Å less for SEPT3. In the latter, PB2 (_162_RKKR_165_) makes well defined electrostatic interactions with the polyacidic region (_240_EFDEDLED_247_) including the following salt bridges: Arg162–Glu240, Lys162–Asp247 and Arg165–Glu243. Arg162 (the homologue of Arg124 in SEPT9) is a key residue in forming the canonical open interface and moves dramatically on interface closure [Figs. 7 ▸(*a*) and 7 ▸(*b*)]. These interactions are largely retained in the SEPT3G–GMPPNP complex although there is more structural disorder in this case, particularly within the polyacidic region. Specific interactions are less easily defined in the case of SEPT9G–GTPγS owing to the lower resolution, but the proximity of PB2 to the polyacidic region as a result of interface closure is clearly evident [Fig. 7 ▸(*c*)]. In the case of the open interfaces the main chain of the polyacidic region is traceable, but there is considerable variation in terms of side-chain disorder.

Surprisingly, both SEPT12G structures (bound to either GDP or GMPPNP) present two types of NC interface within the same filament. This is perhaps the most striking example of the plasticity of the NC interface within the SEPT3 subgroup. Whilst the open interface (NC_1_) is canonical [similar to Fig. 7 ▸(*a*)] the other (NC_2_) is both shifted and closed, causing the α6 helices to be displaced by approximately 7.6 Å parallel to their axes. The interface is therefore asymmetric, in which reciprocal interactions are no longer observed between the two subunits [Fig. 7 ▸(*d*)]. As a result, the interactions between PB2 and the polyacidic region are limited to only one side of the interface and only the NC_1_ (open) interfaces have twofold-symmetry axes perpendicular to the main filament axis (Valadares *et al.*, 2017 ▸). The consequence is a slight loss of filament linearity, leading to the zigzag appearance that can be seen in Fig. 3 ▸(*a*).

## 4. Discussion   

### 4.1. The G interface   

The reason for the apparent redundancy of human septin genes and their division into different subgroups is still far from fully understood. By providing a complete compendium of structures of the SEPT3 subgroup bound to both GDP and GTP analogues, we provide a means of identifying common features between them which are likely to explain their expected capability to substitute for one another at equivalent positions within heteromeric octamers (Kinoshita, 2003 ▸). We define residues that are conserved in all SEPT3-subgroup members but are not present in any other human septin to be ‘characteristic’ of the subgroup, and we will use this term in much of the following discussion.

The G interface observed in all structures reported here is likely to be promiscuous (nonphysiological), at least within the context of hetero-octameric particles. Rather, the current model for the oligomeric assembly predicts that SEPT3-subgroup members will form G interfaces with SEPT7 (Supplementary Fig. S3). Nevertheless, the G interfaces observed here are remarkably similar to those reported previously for other septins. One notable feature of the SEPT3 subgroup is the presence of the characteristic residue Thr282 (SEPT3) from the β-meander. This replaces a tyrosine that is present in all other subgroups that reaches across the interface to interact with the G4 GTPase motif of its neighbour. The lack of this tyrosine is likely to explain why all SEPT3-subgroup members were purified as monomers in this work. Furthermore, it has been shown that reintroduction of the tyrosine into SEPT3 by mutagenesis induces dimerization (Macedo *et al.*, 2013 ▸).

Notwithstanding the existence of characteristic residues, there may also be specific features of a particular SEPT3-subgroup member which distinguish it from the others. These may be related to specific roles in particular hetero-oligomeric complexes. For example, a unique feature of SEPT12 is the presence of an Arg in the well conserved G4 motif, which becomes _194_ARAD_197_ instead of AKAD as observed in all other human septins. The side chain of Arg195 stacks over one side of the guanine base together with Arg266 on the other side. The ARAD sequence generates a recognition site for a PKA protein kinase which phosphorylates Ser198 (Shen *et al.*, 2017 ▸). This serine is also specific for SEPT12, and the consequence of phosphorylation is the dissociation of Sept12 from the SEPT7–SEPT6–SEPT2/4 heterocomplex by destabilizing the SEPT12–SEPT7 G interface (Shen *et al.*, 2017 ▸).

The SEPT12 structures described here shed light on this phenomenon. The two Ser198 residues lie close to the twofold axis relating the two monomers, with their C^β^ atoms separated by only ∼4.3 Å. The side chain of Arg195 forms three hydrogen bonds to main-chain O atoms of Gly59 of the same subunit together with Pro167 and Ser198 from the other subunit. The formal charge on the arginine therefore appears to be compensated by the partial charges of the dipoles associated with three peptide groups (Quiocho *et al.*, 1987 ▸). These interactions would be expected to be retained by the Lys of SEPT7 at the physiological SEPT12–SEPT7 interface. Given the density of interactions across the interface, the introduction of the large negative phosphoryl moiety on Ser198 of SEPT12 would generate steric hindrance, particularly with Thr198 of SEPT7, thereby destabilizing the interface and releasing SEPT12 from the complex. Such fine regulation of septin–septin contacts is thus likely to be a means by which the formation of oligomers, filaments and higher order complexes are regulated *in vivo* (Shen *et al.*, 2017 ▸).

### 4.2. The plasticity of the NC interface and GTP hydrolysis   

There is still much to be learnt about how the interaction between guanine nucleotides and septins is coupled to filament assembly, bundling and downstream events. Indeed, it is often difficult to separate the effects of binding from those of hydrolysis (Abbey *et al.*, 2019 ▸). At the very least, it has been well established that nucleotide binding is necessary for the correct assembly of the G interfaces, and hydrolysis appears to play an important role in this process (Zent & Wittinghofer, 2014 ▸; Weems & McMurray, 2017 ▸). Furthermore, many reports, in different organisms, have shown phenotypical alterations using GTP-binding or GTP-hydrolysis mutants (Hanai *et al.*, 2004 ▸; Kinoshita *et al.*, 1997 ▸; Sirajuddin *et al.*, 2009 ▸; Versele & Thorner, 2004 ▸; Weems *et al.*, 2014 ▸). For example, in the case of the testis-specific SEPT12, mutants that affect GTP binding and hydrolysis resulted in distinguishable phenotypes in terms of sperm morphology and motility, but were both associated with infertility (Kuo *et al.*, 2012 ▸; Kuo *et al.*, 2015 ▸). Finally, polymerization and the assembly of higher-order structures may well be coupled to membrane association in a way that guarantees the generation of productive complexes (Bertin *et al.*, 2010 ▸; Bridges *et al.*, 2014 ▸; Field *et al.*, 1996 ▸).

The crystal contacts observed here between two identical copies of members of the SEPT3 subgroup form a physio­logical NC interface which occupies a prominent position at the centre of the octameric particle (Supplementary Fig. S3). In all crystal structures solved to date it is the only interface to show any significant degree of plasticity [Fig. 3 ▸(*c*)]. This can best be described with reference to the GDP and GTPγS complexes formed with SEPT9GC. On soaking pre-formed crystals of the GDP complex with excess GTPγS not only was the nucleotide substituted (Supplementary Fig. S4) but the filaments also shrank as a result of the closure of the NC interfaces. This led to a rearrangement of the conserved charged residues which make up the canonical open interface (Fig. 7 ▸). Particularly notable is that closure results in bringing the PB2 region of one monomer into close proximity with the polyacidic region of its neighbour.

**Figure 8:**
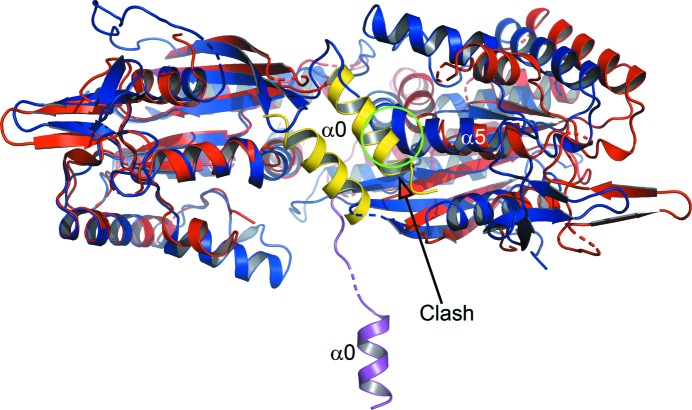
The NC interface and helix α0 (PB1). Superposition of an NC-interface dimer of SEPT2 (PDB entry 2qa5; red) with SEPT3α0G–GDP (PDB entry 4z54; blue). The overlay has been performed on only one of the two subunits (left). Helix α0 of SEPT2 (yellow) is buried within the open NC interface. If the interface were to close such as to occupy a position equivalent to that shown for SEPT3 (closed conformation) a steric clash would occur between α0 and α5 of the neighbouring subunit (green circle). As a consequence α0 would be expelled from the interface. Shown in purple is the position it occupies in the *B* chain of SEPT3α0G–GDP.

However, interface closure would appear to have a second and possibly more dramatic consequence. This concerns the polybasic region corresponding to helix α0 (PB1), which is known to be associated with membrane binding (Zhang *et al.*, 1999 ▸). Once the subunits close, this would be unable to remain in its conventional position anchored within the NC interface. Fig. 8 ▸ shows a superposition of the structure of an NC dimer of SEPT2 (open conformation, PDB entry 2qa5), including helix α0 (in yellow), with that of SEPT3α0G–GDP (closed). The latter is the only structure where we were able to obtain crystals using a construct which includes α0. It now becomes apparent why the α0 helix occupies such a distinct position in the SEPT3α0G structure. It could not reside in a position equivalent to that seen in SEPT2 since this would lead to steric hindrance, principally with helix α5 of the neighbouring monomer. On exposure, α0 gains considerable conformational freedom, so much so that it is only observed in one of the two subunits of the SEPT3G–GDP structure reported here, and that owing to fortuitous crystal packing.

**Figure 9:**
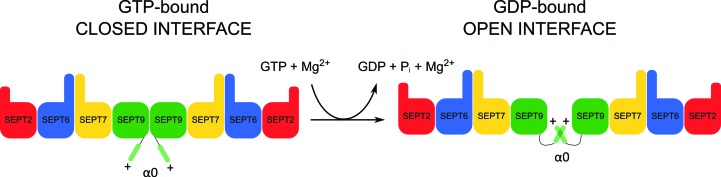
Schematic model of how GTP hydrolysis is coupled to membrane association. The arrangement of the septin octameric complex is shown, in which the SEPT9 homodimeric NC interface, at the centre of the rod, is shown closed (left) and open (right). The corresponding positions for α0, with its positive charge, are exposed and hidden, respectively. The former is expected to favour membrane association.

The structures of SEPT9GC therefore suggest a mechanism by which GTP binding and hydrolysis could be coupled to membrane association (Fig. 9 ▸). On binding GTP the interface would be expected to be closed and the α0 helix exposed, permitting membrane binding, presumably involving PIP2 (Bertin *et al.*, 2010 ▸; Zhang *et al.*, 1999 ▸). Upon hydrolysis, the interface opens, permitting α0 to fold into the NC interface in proximity to the polyacidic region, and thereby disengage from the membrane. Interestingly, ADP-ribosylation factors (Arfs) also show a nucleotide-dependent conformational change leading to the exposure of an N-terminal α-helix known to promote membrane association (Pasqualato *et al.*, 2002 ▸). Like septins, Arfs are also small GTP-binding proteins that are involved in a series of similar processes including membrane-trafficking pathways (D’Souza-Schorey & Chavrier, 2006 ▸).

It is curious to note that the only two crystal structures of constructs which include α0 described to date (PDB entries 2qag and 2qa5) clearly show the helix hidden within the NC interface, with the majority of the basic residues pointing inwards. With hindsight it seems obvious that this could not possibly be the conformation relevant for membrane association. Something is amiss. By contrast, the exposed conformation reported here appears to be ideal for membrane binding via its polybasic sequence. However, this requires a large conformational change. This is likely to be owing to the presence of a highly conserved glycine residue (Gly21 in SEPT9 and Gly59 in SEPT3) which follows the polybasic sequence and which would be able to assume variable but allowable main-chain torsion angles. This residue has been highlighted previously as being characteristic of septins in general (Pan *et al.*, 2007 ▸), and we are now able to provide a structural justification for its conservation. In this context, it is of interest to note that for yeast septins it has been speculated that conformational changes associated with GTP binding, hydrolysis and filament assembly may expose residues for membrane association consistent with what we observe here (McMurray *et al.*, 2011 ▸; Weems *et al.*, 2014 ▸).

It is reasonable to ask why we do not see the same nucleotide-dependent variation at the NC interface in the case of SEPT3 and SEPT12. The structures of SEPT3 bound to either nucleotide are found to have closed NC interfaces, whilst those of SEPT12 present both open and shifted interfaces within the same crystal structure. The latter strongly suggests that the interactions at the interface are fragile and can be readily tipped from one free-energy minimum to another as the result of packing forces. Distinguishing between crystal artefacts and genuine conformational changes is a well known problem in protein crystallography. As a consequence, it is possible that SEPT3 and SEPT12 could display the same behaviour as SEPT9 but have become trapped by lattice contacts. Alternatively, there may be intrinsic differences between the behaviour of the three SEPT3-subgroup septins which ultimately would be related to their specific functions. For example, it should be recalled that whilst SEPT9 is ubiquitously expressed, SEPT3 and SEPT12 are largely restricted to neural cells and the testis, respectively, where they play specialized roles.

With this proviso, SEPT9 still seems to provide the most reliable data available, simply because the GTPγS complex was obtained by soaking GDP-bound crystals in their own crystallization solution to which the GTP analogue had been added. It would therefore appear that the change in the NC interface must be a direct consequence of the nucleotide exchange, as summarized in the model shown in Fig. 9 ▸. It remains to be established whether SEPT3 and SEPT12 are indeed capable of a similar behaviour.

**Figure 10:**
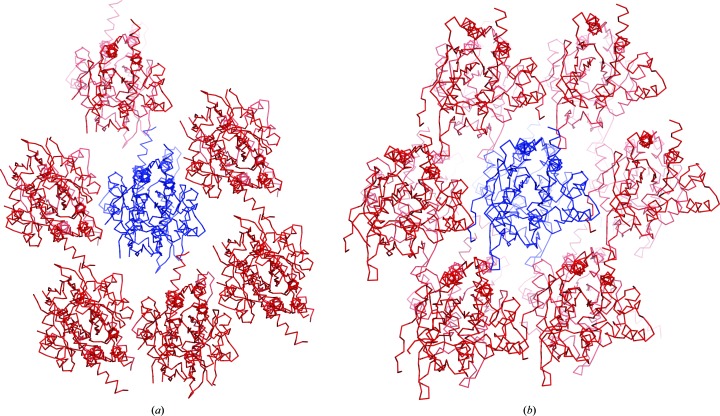
Crystal packing in (*a*) SEPT9GC–GDP and (*b*) SEPT3α0G–GDP. In both cases the view is along the filaments within the crystal. In (*a*) the interaction between the central filament (blue) and its neighbours (red) appears to be fragile owing to relatively few crystal contacts. Presumably, it is the paucity of these contacts which permits the filaments to shrink on soaking with GTPγS. In (*b*) SEPT3α0G–GDP, which has a slightly tighter packing, is shown for comparison.

It is interesting to understand how the soaking experiment was able to generate such a large change to the filaments without destroying the lattice altogether. Fig. 10 ▸ shows how the filaments are arranged within the crystal of the GDP complex used for this experiment. By comparison with that observed in the SEPT3GC–GDP complex, for example, the packing is significantly looser, with many fewer crystal contacts between filaments. This, together with the fact that all filaments lie parallel to one another, presumably facilitates their shrinkage on binding GTPγS without complete destruction of the crystal, albeit with some loss in resolution. In the resulting GTPγS-bound form the filaments appear to be even more loosely packed, leading to apparent gaps in the lattice. However, this is an illusion owing to the very weak and/or uninterpretable electron density in the region of the β-meander.

Our results indicate that the polyacidic region at the entrance to helix α5′ plays two separate roles depending on the conformational state of the NC interface. When the interface is closed the polyacidic region interacts with PB2 of the neighbouring subunit, as observed in the novel interfaces described here (Fig. 7 ▸). On the other hand, the crystal structure of the G domain of SEPT2 (PDB entry 2qa5) shows the polyacidic region to play a role in providing a safe haven for PB1 of helix α0 when the interface is open and the helix is hidden. In this case, a detailed description of the interactions involved is hampered by structural disorder and low resolution.

It is possible that the details of these interactions may not be identical in different septins, and in this respect the SEPT3 subgroup is particularly interesting. In this case, helix α5′ is orientated very differently, lying more parallel to the filament axis (Fig. 6 ▸). This difference compared with other sub­groups is owing to a cluster of ‘characteristic’ residues belonging uniquely to the SEPT3 subgroup, including Pro237, Phe241 and Ile319 (Fig. 6 ▸). The main chain deviates significantly after *cis* Pro241, thereby lifting the polyacidic region, which is further stabilized by the hydrophobic contact between Phe241 and Ile319. This results in bringing PB2 into close proximity with the polyacidic region of the neighbouring subunit in the closed conformation.

The differences that we describe here for α0, α5′ and the polyacidic region, and the plasticity of the NC interface, all suggest that the homotypic interactions formed by SEPT3-subgroup members may be unique. For example, no such variability has been observed at other NC interfaces, be they homotypic or heterotypic, all of which show the canonical open conformation. It is possible, therefore, that only SEPT3-subgroup members possess the capability to open and close the NC interface, with the corresponding sequestering and exposing of the α0 helix. It is tempting to speculate that this plasticity may be related to the lack of a C-terminal coiled coil in the case of this septin subgroup, which may allow adjacent monomers to adopt different relative positions.

SEPT3 members occupy the central position of the octameric rod, analogous to Cdc10 in yeast (Supplementary Fig. S3). It is therefore worthy of note that several experimental approaches have demonstrated that it is the polybasic region of Cdc10 which is the dominant feature in driving the association with PIP2-containing lipid monolayers (Bertin *et al.*, 2010 ▸). Therefore, despite some important differences between the two systems, there may also be useful parallels to be drawn, including the possibility that it is the centre of the oligomeric rod which plays a dominant role in membrane association. It is interesting to note that when comparing the intrinsic helical tendency of peptides corresponding to α0 from representatives of the four subgroups, only the representative of the SEPT3 subgroup (SEPT9) showed a strong α-helical tendency using SRCD spectroscopy (Supplementary Fig. S5) and this was coherent with secondary-structure prediction.

### 4.3. Communication between the interfaces   

The above discussion raises the question of how structural information may be transmitted from one interface to another along the filament, more specifically from the G interface to the NC interface as a result of GTP hydrolysis. A few years ago a potential explanation was offered in the form of β-strand slippage, which was observed in both *Sm*SEPT10 and human SEPT2 (Zeraik *et al.*, 2014 ▸; Valadares *et al.*, 2017 ▸). However, recent evidence suggests that this is probably an artefact which occurs in the case of nonphysiological or ‘promiscuous’ interfaces (Brognara *et al.*, 2019 ▸). Indeed, on comparison of the three sets of complexes reported here, all show the β3 strand to have the same register with respect to its neighbours (β1 and β2) and therefore no β-strand slippage has occurred as a function of the nature of the nucleotide bound to the active site.

**Figure 11:**
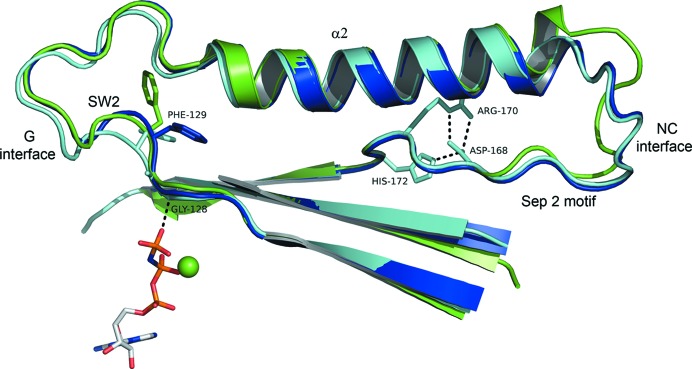
Helix α2 connects the two interfaces. A superposition of the SEPT3G–GMPPNP complex (light blue) with SEPT3α0G–GDP (dark blue) and SEPT9GC–GDP (dark green) in the region of switch II, helix α2 and the sep2 motif. Helix α2 traverses the molecule from one interface to the other. Phe129 (SEPT3 numbering) is shown. All molecules bound to a GTP analogue show this phenylalanine in the buried position (light blue), whilst the GDP complexes show much variation, with examples of all three of the conformations shown. The sep2 motif lies underneath α2 such that it does not make conventional packing contacts with the underlying β-sheet. α2 may be a means to transmit structural information from the G interface to the NC interface once switch II is released after GTP hydrolysis.

Helix α2 is a second conspicuous structural element which runs directly between the two interfaces and is a possible candidate to be the conduit. This is a very prominent feature of septins and is unusual when compared with other small GTPases (Valadares *et al.*, 2017 ▸). Strikingly, it is unable to pack conventionally against the underlying β-sheet owing to an impediment formed by the following loop, which is interposed between the helix and the surface of the sheet. This region corresponds to the so-called sep2 motif (Pan *et al.*, 2007 ▸), the high sequence conservation of which in septins has yet to be explained. The side chains of Asp168, Arg170 and His172 from the beginning of the sep2 motif form a conserved hydrogen-bonded cluster which faces the underside of α2. These features disconnect the helix from packing normally against the hydrophobic core and may give it greater conformational autonomy (Fig. 11 ▸).

Helix α2 is coupled to the G interface, at its N-terminus, via switch II and to the NC interface, at its C-terminus, via PB2. The latter participates in salt bridges in both the open and closed conformations described above. Within the switch II region, all GTP-analogue complexes reported here have Phe129 (SEPT3) or its homologue pointing towards the α2 helix. The lack of the γ-phosphate in the GDP complexes frees up switch II and the homologue of Phe129 is observed in several different conformations in different GDP complexes. This additional conformational flexibility has the potential to perturb the N-terminal region of helix α2 (as seen in SEPT9GC, for example, where the side chain of Cys100 must move out of its way), thereby potentially providing a means to transmit information to the NC interface via the helix itself. In such a way, the energy released by hydrolysis of GTP at the G interface could be transmitted to the adjacent NC interface via a modification of the universal switch mechanism in which the release of the γ-phosphate generates conformational freedom within switch II which is subsequently transmitted via helix α2. Although rather speculative and devoid of detail, this model provides a working hypothesis and may justify the unusual packing of helix α2 and its relationship to the septin-specific motif sep2. No other structural explanation readily presents itself.

In summary, the wealth of information provided by having access to a complete set of crystal structures of the SEPT3 subgroup has allowed us to suggest a dynamic mechanism which couples GTP hydrolysis to membrane association. In the case of SEPT9, direct crystallographic evidence supports this proposal, and the conservation of a series of important structural motifs in all other members of the subgroup suggests that it may be generally applicable. Nevertheless, this remains to be demonstrated (or disproved) for SEPT3 and SEPT12. Furthermore, we speculate that it is the SEPT3 subgroup of septins which plays a dominant role in the association of the septin filament with PIP2-containing membranes, as is the case for Cdc10 in yeast. This raises the intriguing question of how filaments lacking a SEPT3-subgroup member (should they exist) perform their physiological roles.

## Supplementary Material

PDB reference: SEPT3G–GTPγS, 4z51

PDB reference: SEPT3α0G–GDP, 4z54

PDB reference: SEPT9GC–GDP, 5cyo

PDB reference: SEPT9GC–GTPγS, 5cyp

PDB reference: SEPT12G–GMPPNP, 6mq9

PDB reference: 6mqb

PDB reference: SEPT12G–GDP, 6mqk

Supplementary figures. DOI: 10.1107/S2052252520002973/lz5033sup1.pdf

## Figures and Tables

**Table 1:** Protein-expression parameters

	SEPT9GC	SEPT12G
Fragment	279–568	47–320
Expression temperature (°C)	22	18
[IPTG] (m*M*)	0.2	0.2
Lysis buffer	25 m*M* Tris–HCl pH 7.8, 300 m*M* NaCl, 5 m*M* MgCl_2_, 5 m*M* β-mercaptoethanol, 12% glycerol	50 m*M* Tris–HCl pH 8.0, 500 m*M* NaCl, 10% glycerol, 50 m*M* arginine, 50 m*M* glutamic acid, 5 m*M* TCEP
Affinity column	TALON Superflow	Nickel–nitriloacetic acid resin
Activity buffer	25 m*M* Tris–HCl pH 7.8, 300 m*M* NaCl, 5 m*M* MgCl_2_, 8% glycerol	50 m*M* Tris–HCl pH 8.0, 500 m*M* NaCl, 10% glycerol
SEC buffer	20 m*M* HEPES pH 7.5, 300 m*M* NaCl, 5 m*M* MgCl_2_, 12% glycerol, 5 m*M* β-mercaptoethanol	50 m*M* Tris–HCl pH 8.0, 500 m*M* NaCl, 10% glycerol, 50 m*M* arginine, 50 m*M* glutamic acid, 5 m*M* TCEP

**Table 2:** Data-collection and processing statistics

	SEPT3Gα0–GDP	SEPT3G–GMPPNP	SEPT9GC–GDP	SEPT9GC–GTPγS	SEPT12G–GMPPNP	SEPT12G–GMPPNP	SEPT12G–GDP
Detector	PILATUS 6M	PILATUS 6M	ADSC Quantum 315r	ADSC Quantum 315r	PILATUS3 6M	PILATUS3 6M	PILATUS3 6M
Unit-cell parameters							
*a* (Å)	43.41	51.28	57.50	74.59	47.50	41.02	46.96
*b* (Å)	44.55	74.27	78.05	79.16	70.30	91.86	69.25
*c* (Å)	78.95	79.21	77.44	108.22	89.21	151.68	88.23
α (°)	99.29	90.00	90.0	90.0	74.59	490.0	75.45
β (°)	100.77	108.50	105.92	100.4	87.91	90.0	89.46
γ (°)	108.39	90.00	90.0	90.0	78.16	90.0	76.43
Space group	*P*1	*C*2	*P*2_1_	*P*2_1_	*P*1	*C*222_1_	*P*1
Resolution (Å)	41.08–1.83 (1.88–1.83)	75.12–1.86 (1.91–1.86)	55.29–2.03 (2.16–2.03)	50.00–2.89 (3.07–2.89)	46.89–1.86 (1.91–1.86)	45.93–2.12 (2.18–2.12)	45.59–2.19 (2.25–2.19)
X-ray source	I02, DLS	I02, DLS	PROXIMA 1	PROXIMA 1	I24, DLS	I24, DLS	I24, DLS
Wavelength (Å)	0.97949	0.97949	0.9801	0.9801	0.96861	0.96861	0.96861
Multiplicity	2.2 (2.1)	3.6 (3.9)	3.2 (3.1)	3.2 (3.1)	1.9 (2.0)	5.3 (5.4)	3.4 (3.5)
*R*_merge_ (%)	8.7 (24.8)	5.5 (60.1)	3.3 (87.5)	3.2 (78.9)	—	—	—
*R*_p.i.m._ (%)	6.2 (36.5)	3.8 (39.3)	—	—	6.7 (59.2)	6.3 (48.0)	6.8 (60.7)
CC_1/2_	0.993 (0.733)	0.998 (0.715)	0.998 (0.694)	0.995 (0.814)	0.992 (0.572)	0.994 (0.540)	0.995 (0.562)
Completeness (%)	96.5 (94.4)	98.9 (99.7)	99.0 (97.4)	97.6 (95.2)	96.4 (95.2)	99.5 (99.5)	96.7 (96.3)
Reflections	98195 (6766)	85451 (6756)	131992 (20750)	85974 (13284)	170413 (12630)	88807 (6492)	179493 (13725)
Unique reflections	45613 (3289)	23470 (1745)	41929 (6631)	27279 (4266)	88175 (6451)	16639 (1194)	52030 (3910)
〈*I*/σ(*I*)〉	5.4 (1.6)	10.7 (2.3)	11.20 (1.99)	8.57 (1.90)	5.7 (1.4)	7.9 (1.7)	6.9 (1.3)
Reflections used for refinement	45091	23469	41903	27181	86997	16613	52022
*R* (%)	17.16	17.92	18.33	25.22	19.21	20.00	18.43
*R*_free_ (%)	20.19	21.58	22.75	29.74	23.30	24.84	22.86
No. of protein atoms	4341	2009	4183	7117	8709	2158	8643
No. of ligand atoms	56	32	58	133	132	33	116
*B* (Å^2^)	27.20	29.97	42.99	61.23	20.69	38.63	42.2
Coordinate error (Å)	0.21	0.20	0.25	0.40	0.25	0.29	0.31
Phase error (°)	22.95	22.69	24.40	37.14	27.26	25.60	28.39
Ramachandran plot							
Favoured (%)	97.57	96.75	97.68	95.92	97.61	98.12	97.12
Allowed (%)	2.43	2.85	2.32	4.08	2.29	1.88	2.79
Outliers (%)	0.00	0.41	0.00	0.00	0.10	0.00	0.10
All-atom clashscore	3.34	2.5	3.23	5.14	3.02	0.94	3.52
R.m.s.d. from ideal geometry							
Bond lengths (Å)	0.008	0.004	0.004	0.002	0.002	0.002	0.005
Bond angles (°)	1.058	0.821	0.735	0.594	0.571	0.477	0.798
PDB code	4z54	4z51	5cyo	5cyp	6mq9	6mqb	6mqk
