# Supplementary material for: A complete compendium of crystal structures for the human SEPT3 subgroup reveals functional plasticity at a specific septin interface
Source: IUCrJ. 2020 Mar 28;7(Pt 3):462–79. doi: 10.1107/S2052252520002973 (PMC7201284; doi:10.1107/S2052252520002973)
Supplement: Supplementary file 1 [file m-07-00462-sup1.pdf]

IUCrJ

Volume 7 (2020)

Supporting information for article:

A complete compendium of crystal structures for the human SEPT3 subgroup reveals functional plasticity at a specific septin interface

Danielle Karoline Silva do Vale Castro, Sabrina Matos de Oliveira da Silva, Humberto D'Muniz Pereira, Joci Neuby Alves Macedo, Diego Antonio Leonardo, Napoleão Fonseca Valadares, Patricia Suemy Kumagai, José Brandão-Neto, Ana Paula Ulian Araújo and Richard Charles Garratt

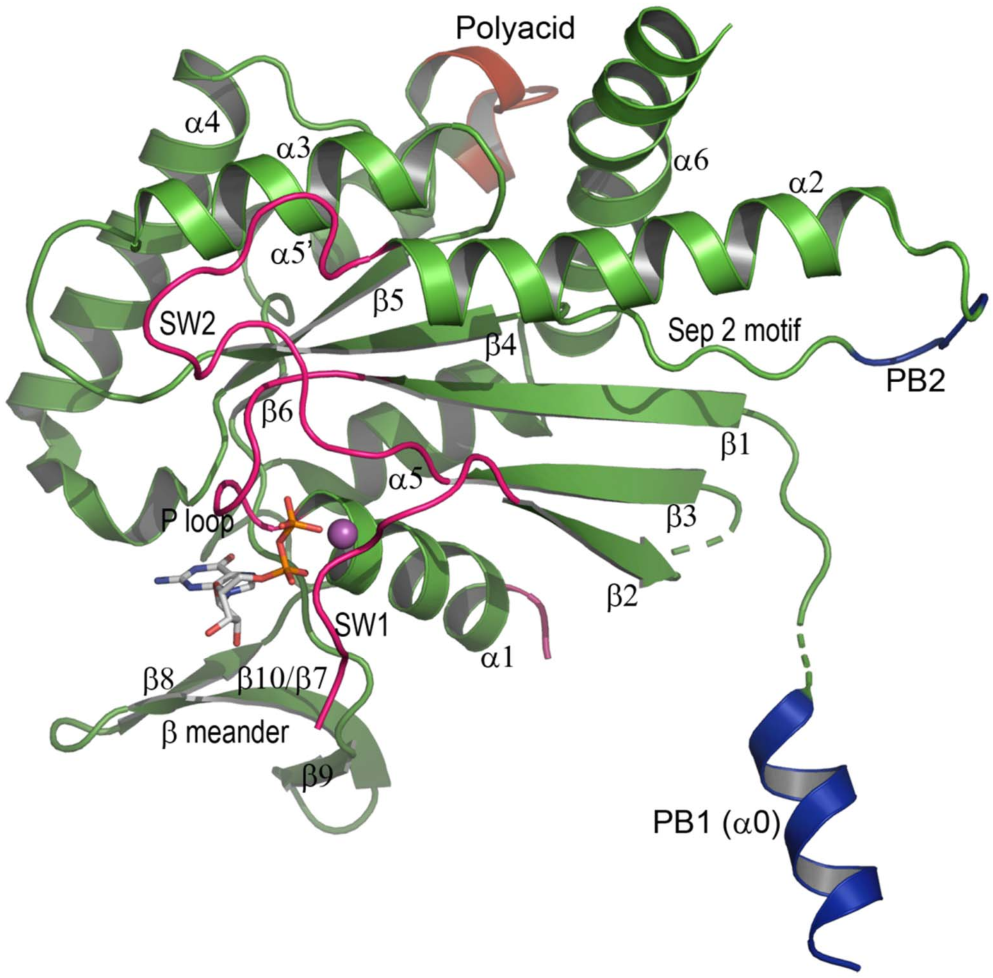

Figure S1 Septin fold nomenclature. Relevant structural features are highlighted on the SEPT3α0G/GDP structure (4Z54) together with the conventional nomenclature for the secondary structure elements. Switches I and II (SW1 and SW2) and the P-loop are shown in purple, the polyacidic region in red and PB1 (α0) and PB2, in blue. The nucleotide is coloured by atom type and the Mg^{2+} ion is shown as a purple sphere.

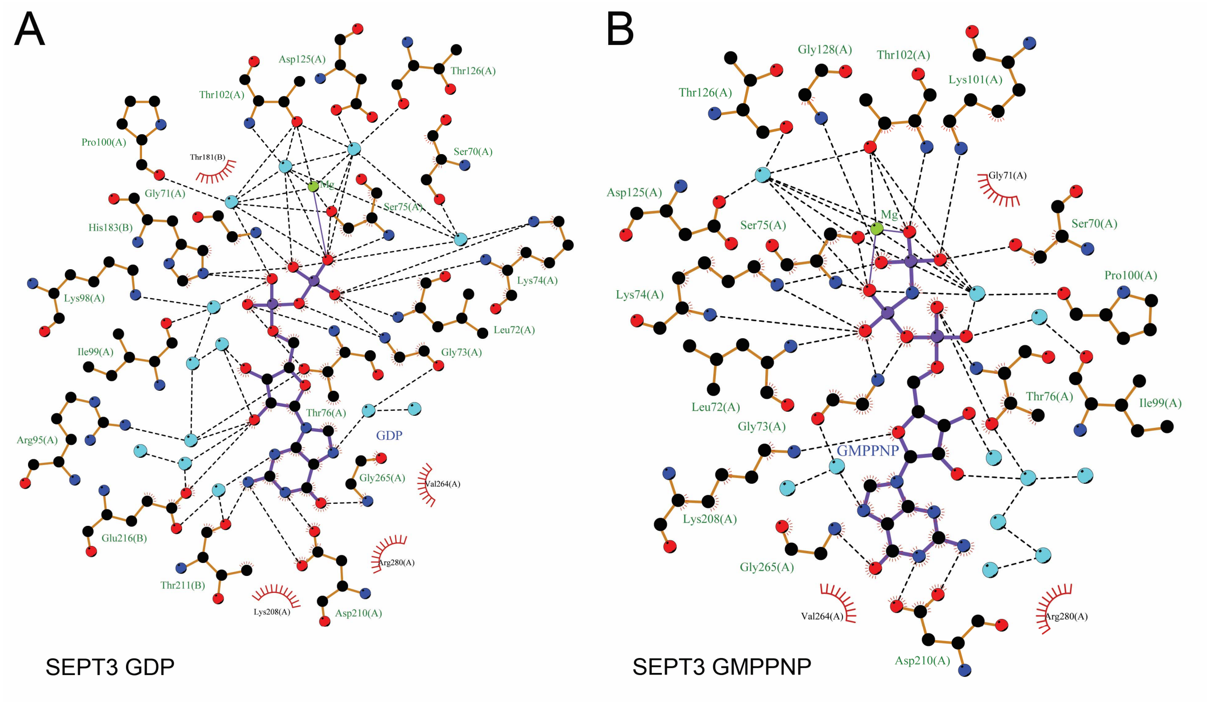

Figure S2 Nucleotide contacts at the active site. Contacts made between active side residues and GDP (left) or GMPPNP (right) are shown for SEPT3. Figures generated with LigPlot+.

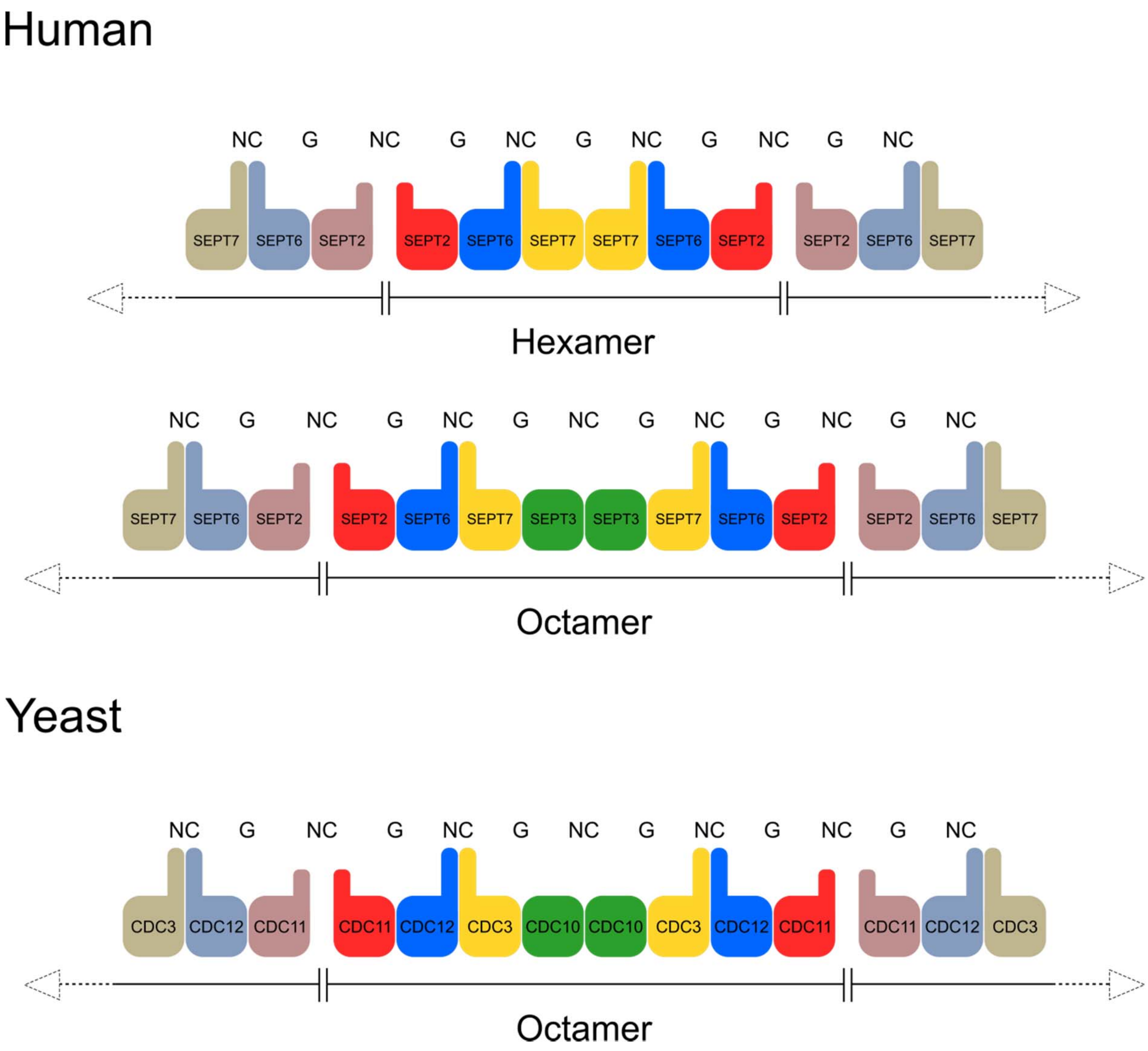

Figure S3 Hexamic and octameric complexes. The current model for the arrangement of both hexameric and octameric rod-like complexes for human septins (above) and yeast octamers (below). The NC and G interfaces are indicated. On polymerization of the complexes to form filaments, new interfaces must form between monomers occupying the terminal positions (red).

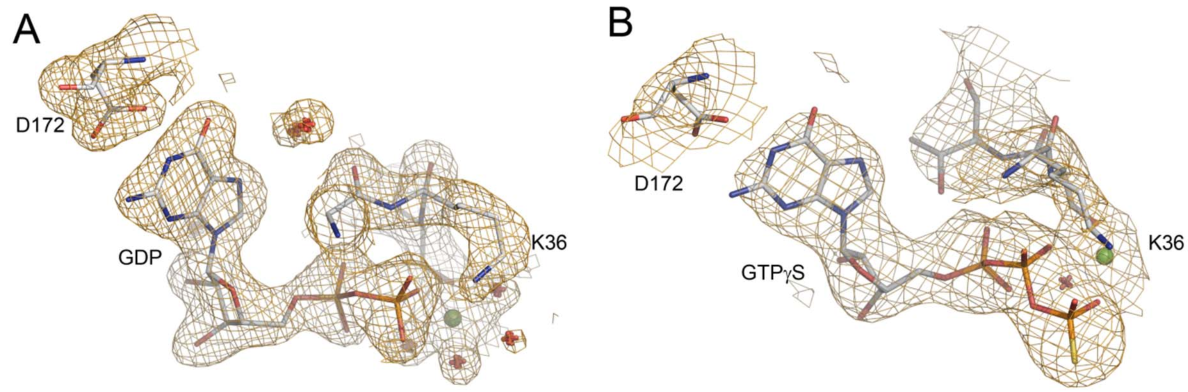

Figure S4 Electron density for the nucleotides in the SEPT9 complexes. Composite omit maps contoured 1.7σ for the region of the nucleotide-binding site for A) the SEPT9GC/GDP complex (5CYO) and B) the SEPT9GC/GTPγS complex (5CYP). In the latter the presence of extra density corresponding to the terminal thiotriphosphate group clearly demonstrates the success of the soaking experiment.

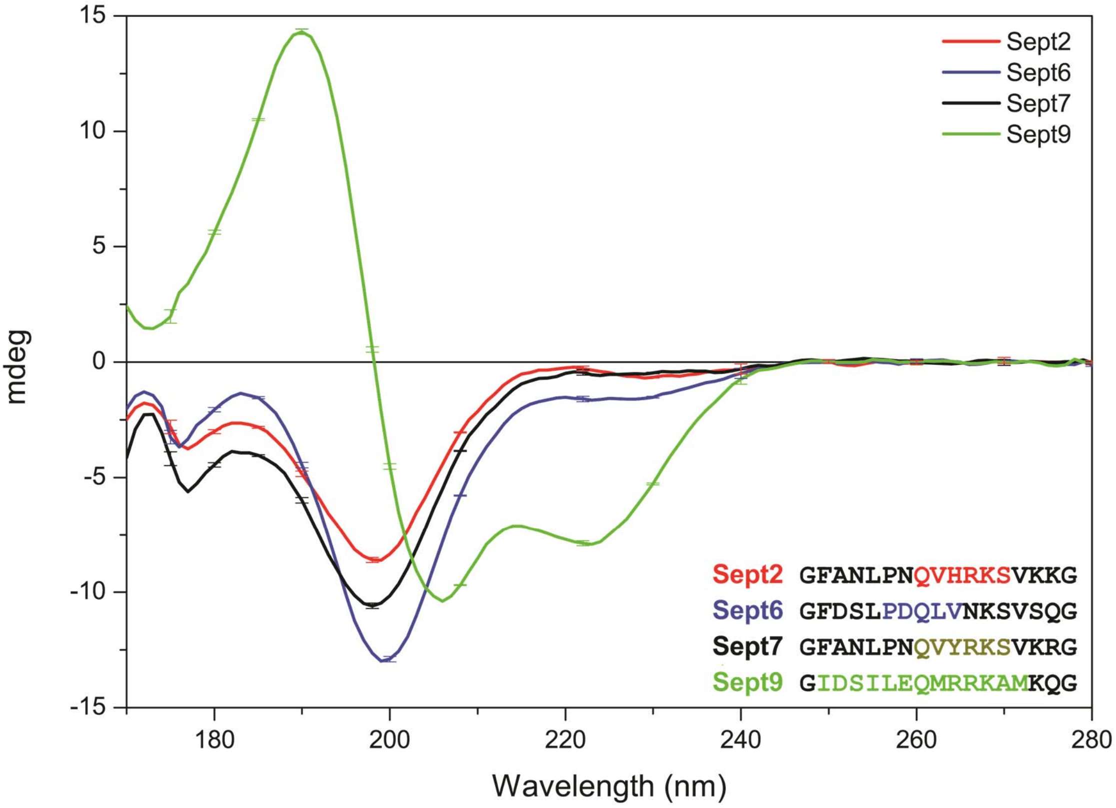

Figure S5 Intrinsic helicity of the PB1 region. SRCD spectra for peptides corresponding to the polybasic region, PB1, for representatives of all four subgroups. Only SEPT9 (representing the SEPT3 subgroup) shows intrinsic helicity in solution, verified by the presence of the positive signal near 190nm and the two troughs at 207nm and 220nm. The inset shows the amino acid sequences of the peptides in which the coloured regions correspond to those predicted to adopt an α-helical conformation. Only SEPT9 shows a significant tendency towards forming an α-helix.
